# Supplementary figures and images for: Bayesian nowcasting with leading indicators applied to COVID-19 fatalities in Sweden
Source: PLoS Comput Biol. 2022 Dec 7;18(12):e1010767. doi: 10.1371/journal.pcbi.1010767 (PMC9762573; doi:10.1371/journal.pcbi.1010767)

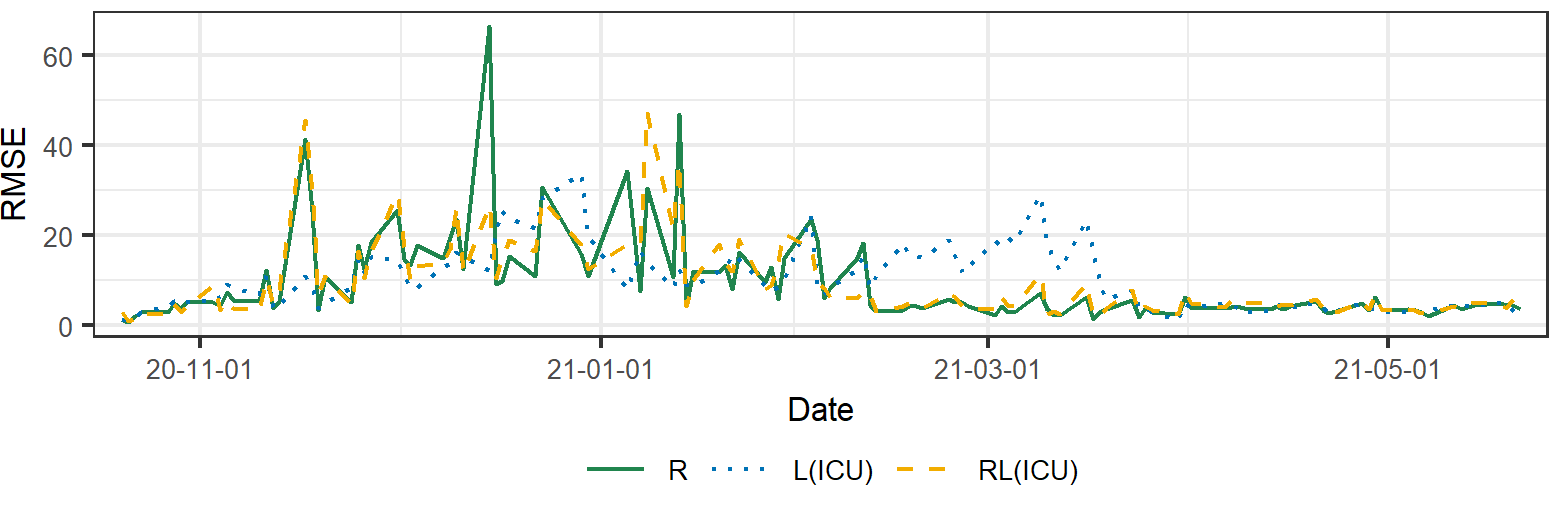

Supplement: S1 Fig — Average RMSE of the last 7 days; T, …, T − 6 for each reporting day T in the evaluation period. (TIF) [file pcbi.1010767.s001.tif]
