# Supplementary material for: Bayesian nowcasting with leading indicators applied to COVID-19 fatalities in Sweden
Source: PLoS Comput Biol. 2022 Dec 7;18(12):e1010767. doi: 10.1371/journal.pcbi.1010767 (PMC9762573; doi:10.1371/journal.pcbi.1010767)
Supplement: S1 Appendix — The priors used in the Bayesian hierarchical models is found in Sec 1. In Sec 2 we show an illustration of the reporting triangle for Swedish COVID-19 deaths. Sec 3 contains detailed information about the nowcasts evaluated at day 2020-12-30 including a figure of the cumulative reporting probability and a table of the evaluation metrics, PI coverage and running times. Detailed results of the estimated regression coefficients of model L(ICU) and RL(ICU) over the evaluation period are found in Sec 4. Sec 5 covers results of including reported cases and the combination of reported cases and ICU admissions as leading indicators. In Sec 6 we show preliminary results of extending the simple random walk into a AR(2) model. Finally, a theoretical comparison of our method and the nowcasting method with covariates by Bastos et al. [19] is found in Sec 7. (PDF) [file pcbi.1010767.s002.pdf]

SI Appendix
Bayesian Nowcasting with leading indicators applied to COVID-19 fatalities in Sweden

Fanny Bergström, Felix Günther, Michael Höhle, Tom Britton.

November 30, 2022

1 Priors

The Bayesian hierarchical nowcasting models used in this paper are specified in Eq (1)-(3) in Sec Materials and Methods. The priors used are found in Table S1.

Table S1. Prior distributions used for the Bayesian hieracical models.

Parameters	
$\sigma \sim \mathrm{N}^+(0, 0.5)$	
$\gamma_d = \mathrm{logit}(p_d / \sum_{i=d}^{D} p_i)$, $p \sim \mathrm{Dir}(1, ..., 1)$	
$\eta \sim \mathrm{N}(0, \sigma_\eta)$, $\sigma_\eta \sim \mathrm{N}^+(0, 0.5)$	
$\phi^{-1} \sim \mathrm{U}[0, 1]$	
$\beta_0 \sim \mathrm{N}(0, 0.2)$	Only model L(M)
$\beta_i \sim \mathrm{N}(0, 0.5)$, $i = 1, ..., k$.	Only model L(M) and RL(M)

N^+ denotes the half-normal distribution. The common priors are the same for model R, L(M), RL(M). Model specific parameters β_0 is used only in model L(M) and β_i, $i = 1, ..., k$, is used only in models L(M) and RL(M).

2 Reported and unreported events

The data used for our analysis comes from the public health agency of Sweden [1]. New reports were available Tuesday throughout Friday (excluding public holidays), which means that there will be gaps in the reporting triangle as it is presented in Fig 1. The reporting triangle for reported Swedish COVID-19 fatalities as of 2020-12-30 is shown in Fig S1A. The green upper triangle are the number of reported reported fatalities and the yellow lower triangle are what is yet unreported. Fig S1B also shows the number of reported (green) and unreported (yellow) number of fatalities for the same period of time where we can see that the reported number of events show a declining trend while the actual number of daily fatalities was at the time increasing. Any empty element in the reporting triangle in Fig S1A means that there was no reporting for this reference day and reporting delay. The diagonal lines of empty elements corresponds to days of non reporting. These days are known to be have zero reporting probability.

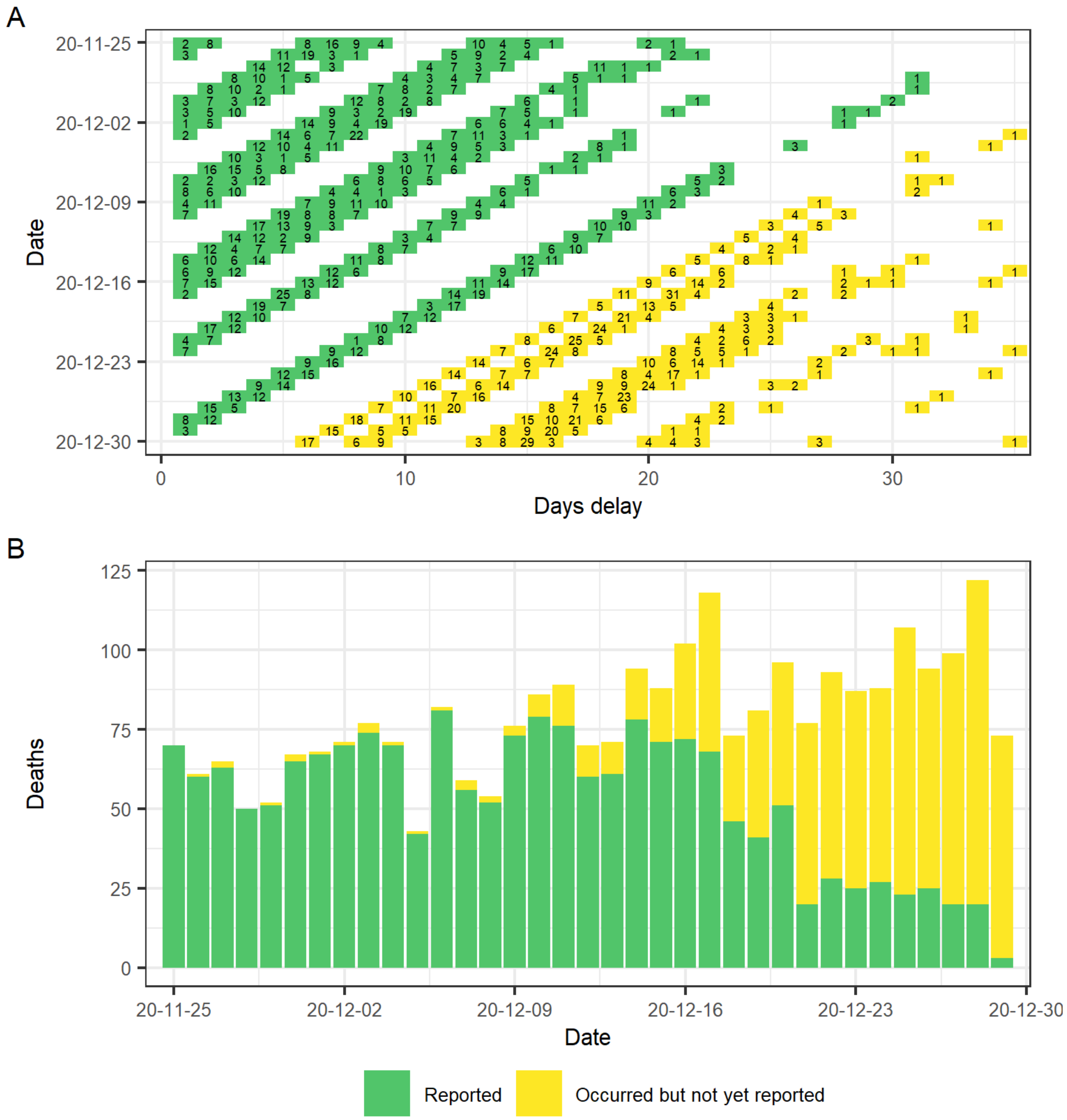

Fig S1. Reporting triangle for Swedish COVID-19 fatalities (A) and reported and unreported fatalities (B) as of 2020-12-30. The figures show what is reported (green) and unreported (yellow). The empty elements of the reporting triangle means that there was no reporting for this day and days reporting delay. The diagonal lines of empty elements represents days when no reporting occurs. Here non-reporting days are Saturday-Monday and public holidays.

3 Evaluation of nowcasts as of 2020-12-30

In this section we present detailed results of nowcasts evaluated at reference day T=2020-12-30.

3.1 Cumulative reporting probability

The cumulative reporting probability is the proportion of reported case fatalities until a certain number of days reporting delay. E.g. if the cumulative probability until 10 days reporting day is 40%, it means that 40% of the cases will be reported within 10 days. In Fig S2, the cumulative reporting probability is shown for the nowcasts estimates for the three models R, L(ICU), and RL(ICU) estimated with the information available as of 2020-12-30 together with the empirical fraction of reported fatalities (known only in hindsight).

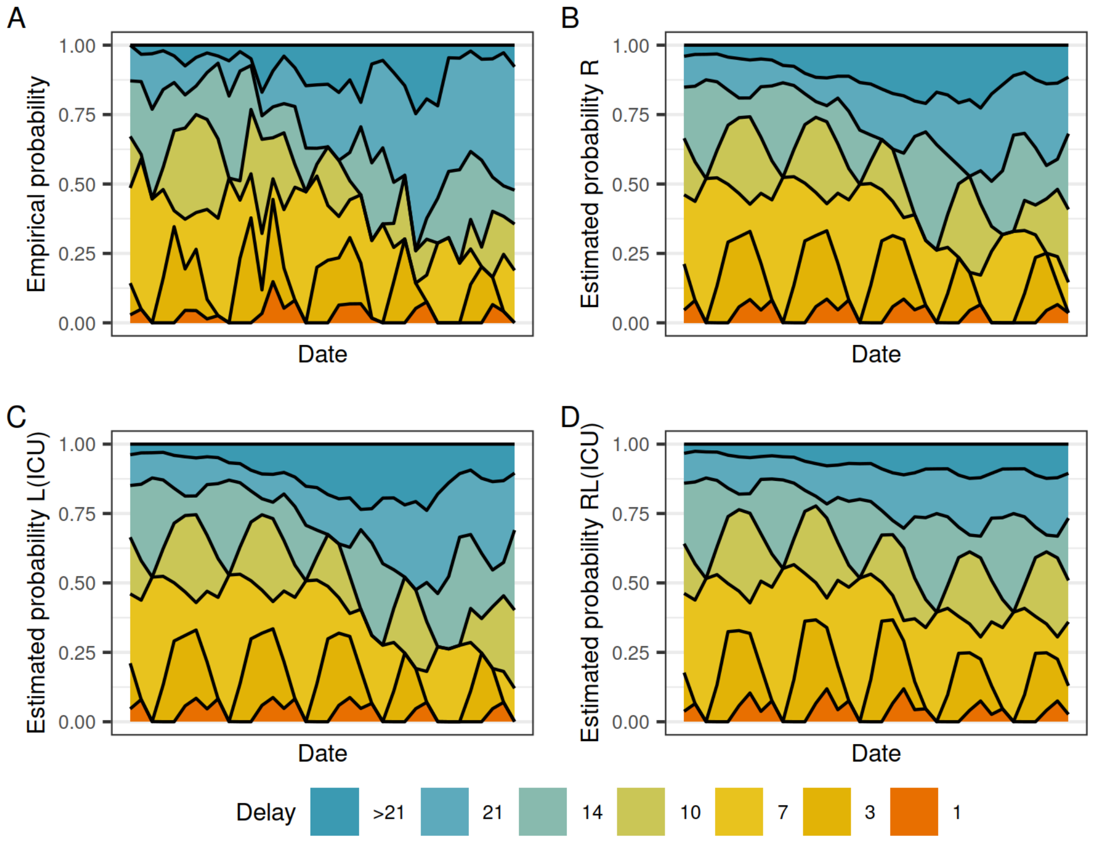

Fig S2. The empirical and estimated cumulative reporting probability of the nowcasts as of T=2020-12-30 for the three models; R, L(ICU), and RL(ICU). For a specific date, the cumulative reporting probability is the fraction of case fatalities occurring on that date reported within a given number of days.

The empirical fraction of the reported fatalities shows a clear weekly pattern but with high irregularities in the reporting from week to week (Fig S2A). One can also note the increase in the reporting delay over time as there is a bigger fraction of longer delays towards the end of the observation window. The three nowcasting models manage to capture the overall weekly pattern as well as the increase in the reporting delay. The cumulative reporting probability is supplementary to Fig 4 in the sense that it also illustrates the estimated reporting delay distribution for the period 2020-11-25–2020-12-30, estimated by the nowcast models with the information available as of 2020-12-30. One can observe that model L(ICU) (Fig S2C) underestimate the reporting delay for the last week of the observation window, leading to an underestimation of the case fatalities for this period. In contrast to model R and RL(ICU) Fig S2B and S2D) that manage to capture the increase in reporting delay to a higher extent. No

case fatalities were reported within the same day for this period.

3.2 Performance metrics and running times

Nowcasts for reference date T=2020-12-30 for model R, L(ICU) and RL(ICU) are shown in Fig 4. In Table S2 we present detailed results in the form of the four evaluation metrics described in Sec Evaluation metrics and running times. We also include results of using reported cases as a second leading indicator in model L(Cases, ICU) and RL(Cases, ICU).

Table S2. Results of retrospective evaluation and running times of nowcasting COVID-19 related fatalities in Ssameweden of one specific reporting date 2020-12-30.

Score	R	L(ICU)	RL(ICU)	L(Cases, ICU)	RL(Cases, ICU)
CRPS	8.49	14.64	8.00	9.15	8.04
logS	4.25	4.84	4.15	4.19	4.13
RMSE	11.57	19.86	11.00	13.86	11.14
Cov. 75% PI	100%	42.9%	100%	85.7%	85.7%
Cov. 90% PI	100%	71.4%	100%	100%	100%
Cov. 95% PI	100%	85.5%	100%	100%	100 %
Running time (sek)	107	62	288	601	374

CRPS is the continuous ranked probability score, logS is the log score, and RMSE denotes the root mean squared error of the posterior median. Additionally, we provide coverage frequencies of 75%, 90% and 95% credibility intervals in the estimation of the daily number of case fatalities. The scores are averaged over nowcasts for day $T, ..., T-6$, T=2020-12-30.

The results for this reporting date entail similar performance of model R and RL(ICU) and RL(Cases, ICU). The scores are similar and all three models have satisfactory PI coverage. Model L(ICU) has low of CRPS compared to model R and RL(ICU) but, as also could be seen in Fig 4, a low PI coverage. A low coverage means that the predictions of this model can not be trusted. Also the RMSE is large which indicate that there is a large deviation of the posterior mean from the observed value. Because of the low PI coverage and high RMSE we consider the performance poor of model L(ICU) for this reporting date. Model L(ICU) was the fastest model with 62 s compared to model R 102 s and model RL(ICU) 288 s. Adding reported cases as a second leading indicator increased running times for both model L and RL and there was no observed improved predictive performance when including the second leading indicator.

These results are just for one single reporting date which does not give a complete picture of the model performance. In Table 1 we average the evaluation metrics over all reporting dates in the evaluation period giving a more solid foundation to base any conclusion about the model performance on. Yet since we illustrate the results for these nowcasts in Fig 4 we also present the corresponding numerical results given by the scoring metrics and the PI coverage.

4 Regression coefficients

The estimated regression coefficients for model L(ICU) and RL(ICU) for each reporting date T in the evaluation period is shown in Fig S3. The regression coefficient is not time-varying for a single estimated nowcast but is reestimated at each reporting date. In the beginning of the evaluation period, the intercept β_0 of model L(ICU) is stable at a low level and the regression coefficient β_1 is increasing as both ICU admissions and case fatalities are increasing. In Jan 2021 when the vaccination is introduced, the association becomes less strong and the β_1 coefficient is decreasing and instead we see an increase of the intercept. When the ICU admissions start to rise

again due to the delta wave, the association also rises, but this time on false premises since the association now is less strong, resulting in the bad performance of this model in the period of 2021-02-15–2021-03-15. Eventually, the new association are captured by the model and the final two weeks of the evaluation period the model has a stable intercept β_0 and a low estimate of β_1.

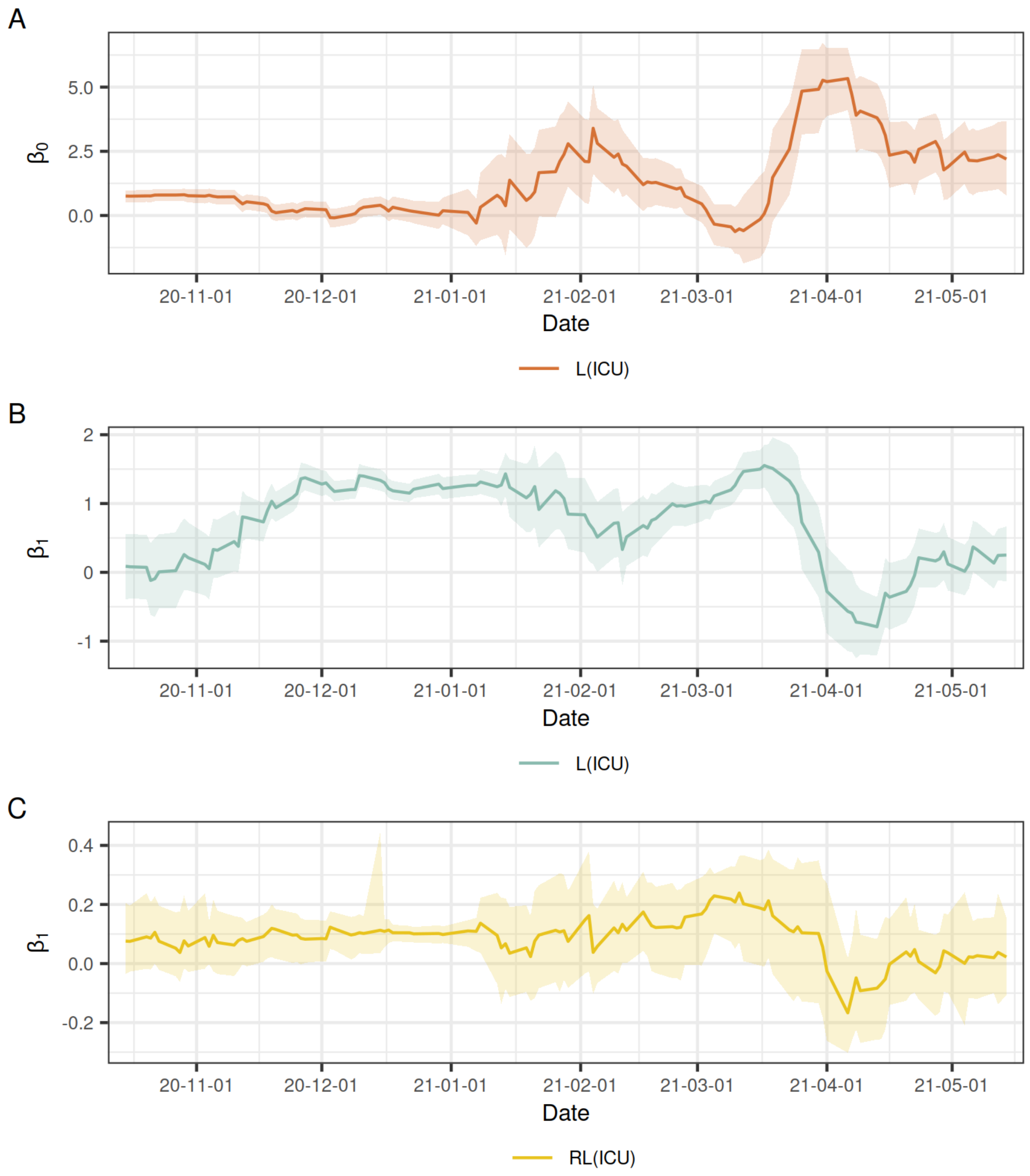

Fig S3. Estimated regression coefficients for L(ICU) and RL(ICU. The solid lines are the median of the posterior predictive distribution of β_0 and β_1 and the shaded areas indicate the equal-tailed point-wise 95% Bayesian prediction interval.

The estimated regression coefficient β_1 of model RL(ICU) (Fig S3C), where the leading indicator is the weekly change in ICU admissions, is more constant until the introduction of

vaccines. After that both ICU admission and fatalities drop, and the estimated association stays positive. During the delta wave, the case fatalities continue to decrease while the ICU admissions start to rise, hence the estimated negative association during April 2021. The final two weeks of the observation period, the median of the posterior distribution of β_1 is centered around zero.

5 Multiple data streams

In addition to using ICU admission as a single leading indicator, we also use reported cases and the combination of reported cases and ICU admissions as leading indicators. In Fig S4, the result of the model RL(Cases) and RL(Cases, ICU) is shown.

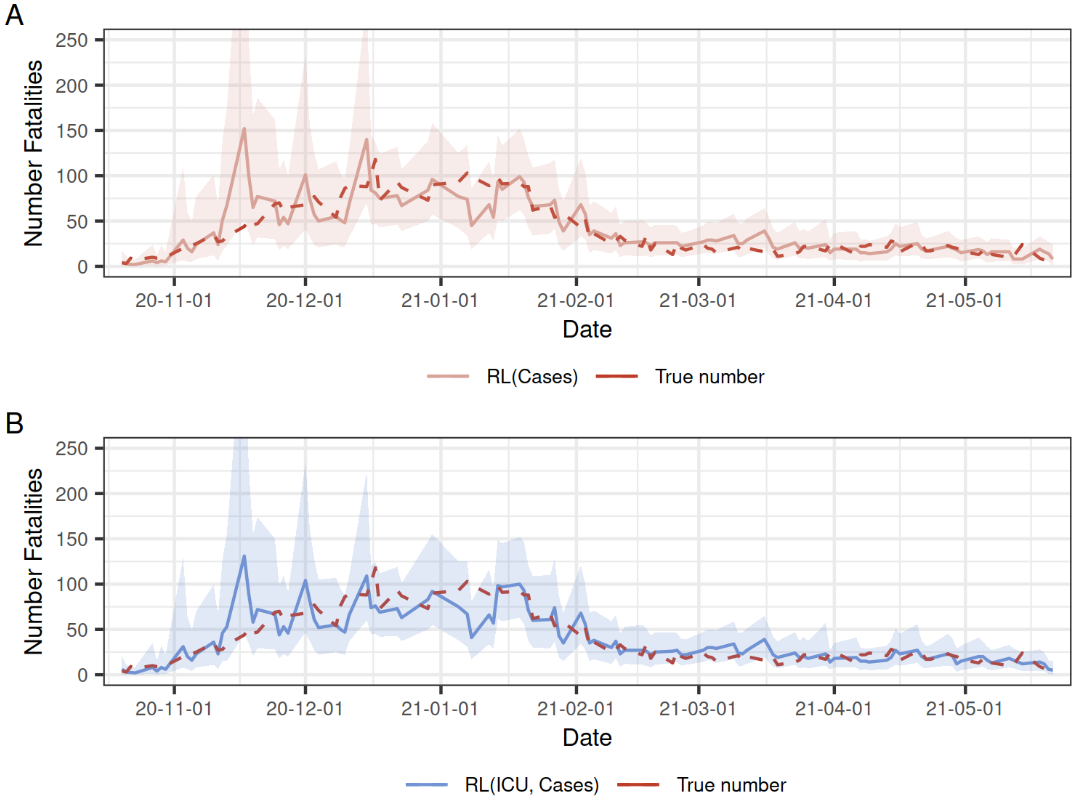

Fig S4. Median of the posterior predictive distribution of $\hat{N}_T$ (solid line) and a 95% PI, and the actual number of fatalities (known in hindsight) for each reporting date T in the evaluation period.

The predictive performance of models RL(Cases) and RL(Cases, ICU) is worse compared to model RL(ICU). The association between the reported cases and the case fatalities appears to be lower than for the ICU admissions, which explains that there is no gain in including the reported cases as a leading indicator. The time series of reported cases heavily depends on the reporting capacity and strategy, as well as the propensity of the general public to get tested, which all has been changing rapidly under the course of the pandemic. The association between the ICU admissions and case fatalities has been more stable throughout the pandemic (with the exception of the introduction of the vaccines in Jan 2021) making the ICU admissions a suitable leading indicator for our application of nowcasting the Swedish COVID-19 case fatalities.

6 AR(2) model for λ_t

We investigated if a autoregressive (AR) model of order 2 would improve the predictive performance of the nowcasts compared to the simple random walk used in model R. In the comparison we used model R with λ_t defined as in Eq (1) and a AR(2) model specified as

$$\log(\lambda_t)|\lambda_{t-1}, \lambda_{t-2}, \beta_1, \beta_2 \sim N(\beta_1 \log(\lambda_{t-1}) + \beta_2 \log(\lambda_{t-2}), \sigma^2), \quad (1)$$

where $t = 2, \ldots, T$ and β_i coefficient of $\log(\lambda_t - i)$. We denote this model R2.

Models R and R2 are evaluated at 11 reporting dates (every 10th reporting date in the evaluation period) for this comparison. The numerical results of the scores and PI coverage are found in Table S3.

Table S3. Results of retrospective evaluation of nowcasting COVID-19 related fatalities in Sweden of the random walk model R and a second order random walk model R2.

Score	R	AR2
CRPS	5.45	6.53
logS	3.29	3.32
RMSE	7.75	9.06
Cov. 75% PI	85.7%	80.5%
Cov. 90% PI	96.1%	90.9%
Cov. 95% PI	98.7%	97.4%

Scores and PI coverage for model R and R2. The evaluation metrics are averaged over nowcasts for day $T - 6, \ldots, T - 0$ for the 11 various reporting dates T.

For our application it seams that a the AR model for λ_t does not improve the predictive performance of the nowcasting but we do not exclude that this could be worth further exploring.

7 Comparison to Bastos et al. (2019)

In this section, we compare our proposed Bayesian nowcasting model with a popular alternative nowcasting approach based on log-linear models that is strongly related to the so-called *chain-ladder* method for loss reserving [2]. The Bayesian nowcasting model used here and elsewhere [3, 4, 5] employs the approach of splitting the nowcasting problem into two submodels, one for the expected total number of cases with reference time t, $E(N_t) = E(\sum_{d=1}^{D} n_{t,d}) = \lambda_t$, and the second for characterizing the reporting delay $p_{t,d} = P(\text{reporting delay} = d|\text{reference time} = t)$, conditional on the reference time t. Those two submodels are then combined to specify the likelihood of the observed data, the entries of the reporting triangle $n_{t,d}$, via their expected value $E(n_{t,d}) = \lambda_t \times p_{t,d}$ and all parameters are jointly estimated via MCMC. In contrast, the chain-ladder based models directly formulate a log-linear model for the entries of the reporting triangle, $E(n_{t,d}) = \lambda_{t,d}$. In the simplest case, this log-linear model is of the form

$$log(\lambda_{t,d}) = \mu + \alpha_t + \beta_d. \quad (2)$$

In this model α_t represents (multiplicative) changes in the expected case counts over time in a non-parametric way, and the parameters β_d characterize a time-constant delay distribution. Variations and extensions of this model have been used already early for monitoring infectious disease counts, e.g., by Zeger et al. [6] using a spline for modeling time trends in the overall case counts instead of the non-parametric effects α_t. Recently, Bastos et al. [7] proposed an extended Bayesian version of the chain-ladder model, that also includes *time-delay interaction terms* as

well as seasonal terms and allows the incorporation of covariates in the log-linear model. As this Bayesian chain-ladder model shows many similarities with our hierarchical Bayesian model with leading indicators, we set out to compare them in more detail.

7.1 Model without leading indicators

7.1.1 Time-constant delay distribution

We start the model comparison based on a simple nowcasting model assuming a time-constant delay distribution. In the framework of Bastos et al. [7] we can formulate this model by specifying the expected number of cases with reference time t and reported with a delay of d time steps, $E(n_{t,d}) = \lambda_{t,d}$ as in the standard chain-ladder model in Eq (2), as

$$log(\lambda_{t,d}) = \mu + \alpha_t + \beta_d.$$

For estimation of the parameters in a Bayesian context, Bastos et al. [7] propose random-walk prior distributions that represent a smootheness asumption: $\alpha_t \sim N(\alpha_{t-1}, \sigma_\alpha)$, $\beta_d \sim N(\beta_{d-1}, \sigma_\beta)$, and specify some identifiability constraints.

To compare this model with ours, we derive the expected number of total cases with reference time t, i.e., $N_t = \sum_{d=0}^{D} n_{t,d}$. In the Bastos et al. [7] model this corresponds to

$$\begin{aligned} E(N_t) = \lambda_t = E(\sum_{d=0}^{D} n_{t,d}) = \sum_{d=0}^{D} E(n_{t,d}) = \sum_{d=0}^{D} \lambda_{t,d} \\ = \sum_{d=0}^{D} e^{\mu+\alpha_t+\beta_d} \\ = e^{\mu+\alpha_t} \times \sum_{d=0}^{D} e^{\beta_d} \end{aligned}$$

To interpret the first-order random walk prior for α_t in the context of the expected number of cases with reference time t, λ_t, we solve for α_t:

$$\begin{aligned} \alpha_t &= log\Big(\frac{\lambda_t}{e^{\mu} \times \sum_{d=0}^{D} e^{\beta_d}}\Big) \\ &= log\Big(\frac{\lambda_t}{c}\Big) = log(\lambda_t) - log(c). \end{aligned}$$

A first order random walk prior for $\alpha_t \sim N(\alpha_{t-1}, \sigma_\alpha)$ implies

$$\begin{aligned} \alpha_t &= \alpha_{t-1} + \epsilon_t \\ \Leftrightarrow log(\lambda_t) - log(c) &= log(\lambda_{t-1}) - log(c) + \epsilon_t, \end{aligned}$$

with $\epsilon_t \sim N(0, \sigma_\alpha)$, i.e.,

$$log(\lambda_t) \sim N(log(\lambda_{t-1}), \sigma_\alpha).$$

This corresponds to the prior distribution for λ_t that we use in our baseline model (called model R in the manuscript).

7.1.2 Time-varying delay distribution

Our hierarchical Bayesian model allows us to combine the first-order random walk model for $log(\lambda_t)$ with a separate discrete time hazard model for the reporting delay that can be flexibly specified to account for changes in the reporting delay distribution over time.

One way to achieve changes in the reporting delay distribution over time in the log-linear model framework of Bastos et al. [7] is to include *time-delay interaction terms* $\gamma_{t,d}$ into the model:

$$log(\lambda_{t,d}) = \mu + \alpha_t + \beta_d + \gamma_{t,d}. \tag{3}$$

Note that Bastos et al. [7] propose a specific prior for modelling the $\gamma_{t,d}$'s that they refer to as a first-order random walk on each *delay-column*, i.e., $\gamma_{t,d} \sim N(\gamma_{t-1,d}, \sigma_\gamma)$, but the following derivations are independent of this prior.

Based on Eq (5), one can derive the expected total number of cases with reference time t as:

$$\begin{aligned} E(n_t) = \lambda_t = E(\sum_{d=0}^{D} n_{t,d}) &= \sum_{d=0}^{D} E(n_{t,d}) = \sum_{d=0}^{D} \lambda_{t,d} \\ &= \sum_{d=0}^{D} e^{\mu+\alpha_t+\beta_d+\gamma_{t,d}} \\ &= e^{\mu} \times e^{\alpha_t} \times \sum_{d=0}^{D} e^{\beta_d} \times \sum_{d=0}^{D} e^{\gamma_{t,d}}, \end{aligned}$$

and we can solve for α_t as:

$$\begin{aligned} \alpha_t &= log\Big(\frac{\lambda_t}{e^{\mu} \times \sum_{d=0}^{D} e^{\beta_d} \times \sum_{d=0}^{D} e^{\gamma_{t,d}}}\Big) \\ &= log\Big(\frac{\lambda_t}{c \times \sum_{d=0}^{D} e^{\gamma_{t,d}}}\Big) = log(\lambda_t) - log(c) - log(\sum_{d=0}^{D} e^{\gamma_{t,d}}). \end{aligned} \tag{4}$$

The random walk prior for α_t corresponds then to

$$\begin{aligned} &\alpha_t = \alpha_{t-1} + \epsilon_t \\ \Leftrightarrow\ &log(\lambda_t) = log(\lambda_{t-1}) + log(\sum_{d=0}^{D} e^{\gamma_{t,d}}) - log(\sum_{d=0}^{D} e^{\gamma_{t-1,d}}) + \epsilon_i. \end{aligned} \tag{5}$$

The model-based expected total number of cases with reference time t, $E(n_t) = \lambda_t$, depends therefore not only on the expected total cases of time $t-1$, but also on parameters characterizing differences in the reporting delay distribution of consecutive time points. There is no clear separation between a model for the time trend in total counts and the (time-varying) reporting delay distribution.

7.2 Model with leading indicator

We are now interested in modelling the expected number of cases with reference time t based on a (log-linear) model with a leading indicator as covariate. For this, we assume that we have some data on a covariate x_t available, which is supposed to be used in modeling λ_t. When the goal of the nowcasting is to make inference about the expected number of fatalities at day t, x_t could be the (smoothed) number of reported hospitalizations at day $t-l$, where l is the average time between hospitalization and death to capture the time-lag between the leading indicator and the time-series of interest in the nowcasting application.

7.2.1 Time-constant delay distribution

Bastos et al. [7] argue that it is straightforward to include covariates in the log-linear model for the entries of the reporting triangle. In principle, those covariates can be specific for each entry

(i.e., a covariate $x_{t,d}$, for example when being interested in estimating a week-day effect for a reporting day $t+d$, or specific for the reference time t. The latter is what we are interested in when using a leading indicator x_t for modeling the overall number of cases with reference time t. Without additional *time-delay interaction terms* or seasonal effects, this model corresponds to

$$log(\lambda_{t,d}) = \mu + \alpha_t + \beta_d + \delta x_t,$$

where there are still first-order random walk priors used for the parameters α_t, and β_d. For the expected total number of cases with reference time t, we can derive:

$$\begin{aligned}
\lambda_t &= \sum_{d=0}^{D} e^{\mu+\alpha_t+\beta_d+\delta x_t} \\
&= e^{\mu} \times e^{\alpha_t} \times e^{\delta x_t} \times \sum_{d=0}^{D} e^{\beta_d}, \\
\Leftrightarrow log(\lambda_t) &= \mu + \alpha_t + \delta x_t + log(\sum_{d=0}^{D} e^{\beta_d}) \\
&= \mu^* + \alpha_t + \delta x_t. \qquad (6)
\end{aligned}$$

The first-order random walk prior for $\alpha_t \sim N(\alpha_{t-1}, \sigma_\alpha)$ can also be expressed as $\alpha_t = \alpha_1 + \sum_{k=2}^{t} \epsilon_k$, with prior $\epsilon_k \overset{iid}{\sim} N(0, \sigma_\alpha)$.

We can rewrite Eq (6) therefore as

$$\begin{aligned}
log(\lambda_t) &= \mu^* + \alpha_t + \delta x_t \\
&= \mu^* + \alpha_1 + \sum_{k=2}^{t} \epsilon_k + \delta x_t \\
&= log(\lambda_{t-1}) - \delta x_{t-1} + \epsilon_t + \delta x_t \\
&= log(\lambda_{t-1}) + \epsilon_t + \delta(x_t - x_{t-1}). \qquad (7)
\end{aligned}$$

This corresponds to a first-order random walk assumption:

$$log(\lambda_t) \sim N(log(\lambda_{t-1}) + \delta(x_t - x_{t-1}), \sigma_\alpha),$$

which is close to the model RL*(M)* from our manuscript, when the leading indicator $m_{i,t}$ is defined as first-order differences in the (lagged) leading indicator.

7.2.2 Time-varying delay distribution

Now, we are again interested in the inclusion of *time-delay interaction terms* into the model of Bastos et al. [7] to account for potential changes in the reporting delay distribution over time.

$$log(\lambda_{t,d}) = \mu + \alpha_t + \beta_d + \gamma_{t,d} + \delta x_t.$$

Here

$$\begin{aligned}
\lambda_t &= E(\sum_{d=0}^{D} n_{t,d}) = \sum_{d=0}^{D} E(n_{t,d}) = \sum_{d=0}^{D} \lambda_{t,d} \\
&= \sum_{d=0}^{D} e^{\mu+\alpha_t+\beta_d+\gamma_{t,d}+\delta x_t} \\
&= e^{\mu} \times e^{\alpha_t} \times e^{\delta x_t} \times \sum_{d=0}^{D} e^{\beta_d} \times \sum_{d=0}^{D} e^{\gamma_{t,d}},
\end{aligned}$$

and

$$\alpha_t = log\Big(\frac{\lambda_t}{e^{\mu} \times e^{\delta x_t} \times \sum_{d=0}^{D} e^{\beta_d} \times \sum_{d=0}^{D} e^{\gamma_{t,d}}}\Big)$$
$$= log\Big(\frac{\lambda_t}{c \times e^{\delta x_t} \times \sum_{d=0}^{D} e^{\gamma_{t,d}}}\Big)$$
$$= log(\lambda_t) - log(c) - \delta x_t - log(\sum_{d=0}^{D} e^{\gamma_{t,d}}).$$

The random walk prior for α_t corresponds then to

$$\alpha_t = \alpha_{t-1} + \epsilon_t$$
$$\Leftrightarrow log(\lambda_t) = log(\lambda_{t-1}) + log(\sum_{d=0}^{D} e^{\gamma_{t,d}}) - log(\sum_{d=0}^{D} e^{\gamma_{t-1,d}}) + \delta(x_t - x_{t-1}) + \epsilon_t. \quad (8)$$

In this case, the expected number of total cases with reference time t depends again not only on (differences of) the leading indicator and the expected number of cases for the previous reference time $t-1$, but also on the magnitude of changes in the reporting delay distribution at consecutive time points.

7.3 Summary of the comparison

Our Bayesian hierarchical nowcasting and the extension of chain-ladder nowcasting by Bastos et al. [7] show strong similarities. In a simple model with the assumption of a time-constant delay distribution, the proposed models can be seen as identical with respect to their model for the expected total case counts for a specific reference time t. This is also true for the model including a leading indicator, when the leading indicator is defined in a specific way in the context of the Bayesian hierarchical model. The Bayesian hierarchical model has the advantage that it allows the direct specification of separate models for (i) the expected total case counts with reference time t and (ii) the time-varying delay distribution in an intuitive and well-interpretable way. This allows the user to incorporate knowledge of the reporting process (e.g., weekday effects) directly in the model for reporting delay distribution. The chain-ladder extension is also able to incorporate time changes in the delay distribution into the model, either based on *time-delay interaction terms* or based on time-delay specific covariates. As we have shown with the examples in equations (5) and (8), this can, however, have unexpected implications for the expectation model for the total case numbers per reference time. This makes estimated parameters also hard to interpret. We think that both modelling frameworks provide sufficient flexibility to formulate meaningful nowcasting models in many application situations. In concrete applications, the predictive performance of a model depends on how well specific characteristics of the reporting process (and potentially the relationship between the leading indicator and the target time series) can be represented. Here we think that the better interpretability of the Bayesian nowcasting approach can be very helpful for an adequate model specification.

References

1. Folkhälsomyndigheten. The Public Health Agency of Sweden's COVID-19 data portal; Accessed 2022-03-07. https://www.folkhalsomyndigheten.se/smittskydd-beredskap/utbrott/aktuella-utbrott/covid-19/statistik-och-analyser/.

2. Renshaw AE, Verrall RJ. A stochastic model underlying the chain-ladder technique. British Actuarial Journal. 1988;4(4):903–923.

3. Höhle M, an der Heiden M. Bayesian nowcasting during the STEC 0104:H4 outbreak in Germany, 2011. Biometrics. 2014;70:993–1002.

4. Günther F, Bender A, Katz K, Küchenhoff H, Höhle M. Nowcasting the COVID-19 pandemic in Bavaria. Biom J. 2020;63(3).

5. Abbot S, Lison A, Funk S. Epinowcast: Flexible hierarchical nowcasting. Zendo. 2021. doi:10.5281/zenodo.5637165

6. Zeger SL, See LC, Diggle PJ. Statistical methods for monitoring the AIDS epidemic. Stat Med. 1989;8(1):3–21.

7. Bastos L, Economou T, Gomes M, Villela D, Coelho F, Cruz O, et al. A modelling approach for correcting reporting delays in disease surveillance data. Statistics in Medicine. 2019;38:4363–4377. doi:10.1002/sim.8303.
