# Supplementary material for: Bayesian nowcasting with leading indicators applied to COVID-19 fatalities in Sweden
Source: PLoS Comput Biol. 2022 Dec 7;18(12):e1010767. doi: 10.1371/journal.pcbi.1010767 (PMC9762573; doi:10.1371/journal.pcbi.1010767)
Supplement: Attachment — Submitted filename: PCOMPBIOL-D-22-01107_reviewer.pdf [file pcbi.1010767.s003.pdf]

PLOS Computational Biology

Nowcasting with leading indicators applied to COVID-19 fatalities in Sweden

--Manuscript Draft--

**Manuscript Number:**	PCOMPBIOL-D-22-01107
**Full Title:**	Nowcasting with leading indicators applied to COVID-19 fatalities in Sweden
**Short Title:**	Nowcasting with leading indicators
**Article Type:**	Research Article
**Keywords:**	Nowcasting; Bayesian hierarchical model; COVID-19; epidemic surveillance; infectious disease epidemiology.
**Abstract:**	The real-time analysis of infectious disease surveillance data is essential in obtaining situational awareness about the current dynamics of an adverse health event such as the COVID-19 pandemic. This analysis, of e.g. time-series of reported cases or fatalities, is complicated by reporting delays that lead to underreporting of the number of events for the most recent time points. This can lead to misconceptions by the interpreter, for instance the media or the public, as was the case with the time-series of reported fatalities during the COVID-19 pandemic in Sweden. Nowcasting methods provide real-time estimates of the complete number of events using the incomplete time-series of currently reported events and information about the reporting delays from the past. In this paper we consider nowcasting the number of COVID-19-related fatalities in Sweden. We propose a flexible Bayesian approach, extending existing nowcasting methods by incorporating regression components to accommodate additional information provided by leading indicators such as time-series of the number of reported cases and ICU admissions. We show by a retrospective evaluation that the inclusion of ICU admissions as a leading signal improved the nowcasting performance of case fatalities for COVID-19 in Sweden compared to existing methods.
**Additional Information:**	
**Question**	**Response**
**Financial Disclosure**<br><br>Enter a financial disclosure statement that describes the sources of funding for the work included in this submission. Review the submission guidelines for detailed requirements. View published research articles from *PLOS Computational Biology* for specific examples.<br><br>This statement is required for submission and **will appear in the published article** if the submission is accepted. Please make sure it is accurate.	TB is grateful for financial support from NordForsk, grant no. 105572 (https://www.nordforsk.org/). The computations and data handling was enabled by resources provided by the Swedish National Infrastructure for Computing (SNIC) at HPC2N partially funded by the Swedish Research Council through grant agreement no. 2018-05973 (https://www.snic.se/). The funders had no role in study design, data collection and analysis, decision to publish, or preparation of the manuscript.

Powered by Editorial Manager® and ProduXion Manager® from Aries Systems Corporation

**Unfunded studies**<br>Enter: *The author(s) received no specific funding for this work.*<br><br>**Funded studies**<br>Enter a statement with the following details:<br>• Initials of the authors who received each award<br>• Grant numbers awarded to each author<br>• The full name of each funder<br>• URL of each funder website<br>• Did the sponsors or funders play any role in the study design, data collection and analysis, decision to publish, or preparation of the manuscript?<br>• **NO** - Include this sentence at the end of your statement: *The funders had no role in study design, data collection and analysis, decision to publish, or preparation of the manuscript.*<br>• **YES** - Specify the role(s) played.<br><br>* typeset	
**Competing Interests**<br><br>Use the instructions below to enter a competing interest statement for this submission. On behalf of all authors, disclose any competing interests that could be perceived to bias this work—acknowledging all financial support and any other relevant financial or non-financial competing interests.<br><br>This statement **will appear in the published article** if the submission is accepted. Please make sure it is accurate. View published research articles from *PLOS Computational Biology* for specific examples.	The authors have declared that no competing interests exist.

Powered by Editorial Manager® and ProduXion Manager® from Aries Systems Corporation

**NO authors have competing interests**<br><br>Enter: *The authors have declared that no competing interests exist.*<br><br>**Authors with competing interests**<br><br>Enter competing interest details beginning with this statement:<br><br>*I have read the journal's policy and the authors of this manuscript have the following competing interests: [insert competing interests here]*<br><br>* typeset<br><br>This statement is required for submission and **will appear in the published article** if the submission is accepted. Please make sure it is accurate and that any funding sources listed in your Funding Information later in the submission form are also declared in your Financial Disclosure statement.	
**Data and Code Availability**<br><br>From the time of publication, Authors are required to make fully available and without restriction all data and computational code underlying their findings. Please see our PLOS Data Policy page for detailed policy information, and our Code Sharing page for specific information on code sharing.<br><br>A **Data Availability Statement**, detailing where the data (and code, if applicable) can be accessed, is required at first submission. Insert your Data Availability Statement in the box below. The statement you provide **will be published in the article**, if accepted.<br><br>Please see the Data Reporting section of our submission guidelines for instructions on what you need to include in your Data Availability Statement.	The COVID-19 surveillance data used for the analysis and R-code is openly-available and cited within the manuscript (Materials & Methods).

Powered by Editorial Manager® and ProduXion Manager® from Aries Systems Corporation

If the data and code are all contained in your submission files, please state: *All relevant data are within the manuscript and its Supporting Information files.*<br><br>**PLOS allows rare exemptions to address legal and ethical concerns.** If you have legal or ethical restrictions, please detail these in your Data Availability Statement below for the Journal team to consider.	

Powered by Editorial Manager® and ProduXion Manager® from Aries Systems Corporation

Manuscript
Click here to access/download;Manuscript;Manuscript_Nowcasting_COVID19

Nowcasting with leading indicators applied to COVID-19 fatalities in Sweden

Fanny Bergström[1,*], Felix Günther[1], Michael Höhle[1], Tom Britton[1]

1 Department of Mathematics, Stockholm University, Stockholm, Sweden

* fanny.bergstrom@math.su.se

Abstract

The real-time analysis of infectious disease surveillance data is essential in obtaining situational awareness about the current dynamics of an adverse health event such as the COVID-19 pandemic. This analysis, of e.g. time-series of reported cases or fatalities, is complicated by reporting delays that lead to underreporting of the number of events for the most recent time points. This can lead to misconceptions by the interpreter, for instance the media or the public, as was the case with the time-series of reported fatalities during the COVID-19 pandemic in Sweden. Nowcasting methods provide real-time estimates of the complete number of events using the incomplete time-series of currently reported events and information about the reporting delays from the past. In this paper we consider nowcasting the number of COVID-19-related fatalities in Sweden. We propose a flexible Bayesian approach, extending existing nowcasting methods by incorporating regression components to accommodate additional information provided by leading indicators such as time-series of the number of reported cases and ICU admissions. We show by a retrospective evaluation that the inclusion of ICU admissions as a leading signal improved the nowcasting performance of case fatalities for COVID-19 in Sweden compared to existing methods.

Author summary

Nowcasting methods are an essential tool to provide situational awareness in a pandemic. The methods aim to provide real-time estimates of the complete number of events using the incomplete time-series of currently reported events and the information about the reporting delays from the past. In this paper we consider nowcasting the number of COVID-19 related fatalities in Sweden. We propose a Bayesian approach, extending existing nowcasting methods by incorporating regression components to accommodate additional information provided by leading indicators such as time-series of the number of reported cases and ICU admissions. We use a retrospective evaluation covering the second (alpha) and third (delta) wave of COVID-19 in Sweden to assess the performance of the proposed method. We show that the inclusion of ICU admissions as a regression component improved the nowcasting performance (measured by the CRPS score) of case fatalities for COVID-19 in Sweden by 4.2% compared to an established method.

Introduction

The real-time analysis of infectious disease surveillance data, e.g. in the form of time-series of reported cases or fatalities, is one of the essential components in shaping the response during infectious disease outbreaks such as major food-borne outbreaks or the COVID-19 pandemic. Public health agencies and governments typically use this type of monitoring to assess the disease dynamics and plan and assess the effectiveness of preventive actions [1, 2]. Such real-time analysis is complicated by reporting delays that give rise to *occurred-but-not-yet-reported* events which may lead to underestimation of the complete number of reported events [3]. Fig 1 illustrates the problem with data of Swedish COVID-19-related fatalities as of 2022-02-01. While the reported number of fatalities per day suggested a declining trend, data available two months later [4] revealed that the number at the time was actually increasing.

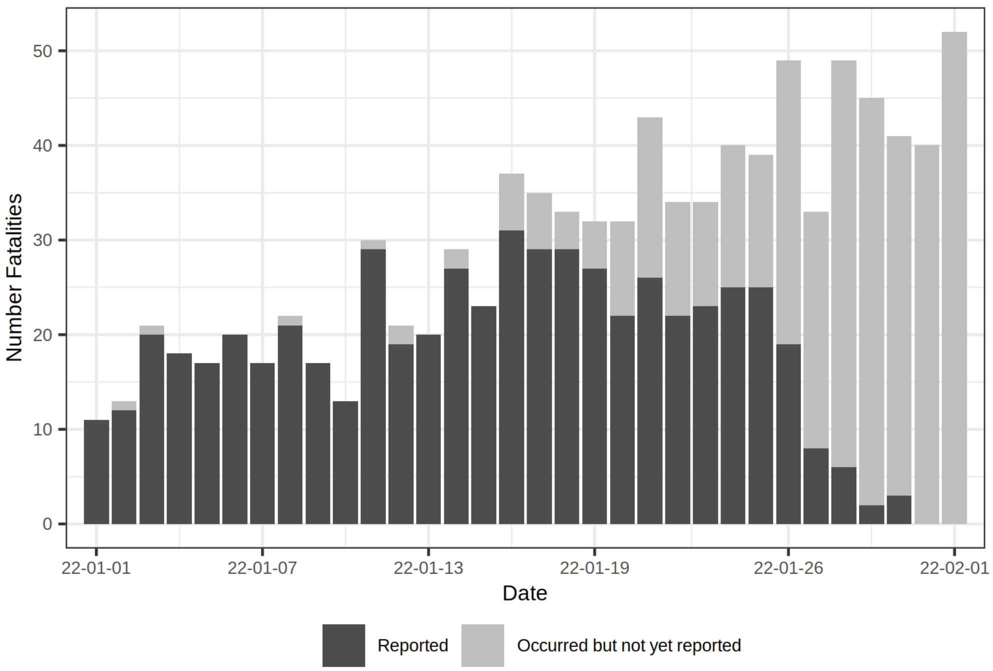

Fig 1. Daily COVID-19 fatalities in Sweden. Reported (black bars) and unreported (grey bars) number of daily fatalities as of 2022-02-01. The reported number of events show a declining trend when in actuality (known in hindsight) it was increasing.

Nowcasting methods [5–7] tackle this problem by providing real-time estimates of the complete number of events using the incomplete time-series of currently observed events and information about the reporting delay from the past. The methods have connections to insurance claims-reserving [8] and its epidemiological applications trace back to HIV modelling [3, 9, 10]. Nowcasting methods has been used in COVID-19 analysis for daily infections [11–13], and fatalities [14–16]. A Bayesian approach to nowcasting, which constitutes the foundation of our method, was developed by Höhle and an der Heiden [6] and later extended by Günther et al. [17] and McGough et al. [7]. Most nowcasting methods are focused on estimating the reporting delay distribution; however, an epidemic contains a temporal dependence since –depending on the mode of transmission– adheres to certain “laws”, e.g. contact behavior which only changes

slowly. Taking the temporal dependence of the underlying disease transmission into account has been shown to improve the nowcasting performance [7, 17]. Another approach to nowcasting, not considering the reporting delay distribution, is to use other data sources that are sufficiently correlated with the time series of interests, e.g. the Machine Learning approach by Peng et al. [18].

Our approach for nowcasting Swedish COVID-19 fatalities is based on a flexible Bayesian hierarchical model that can account for temporal changes in the reporting delay distribution and handle various reporting structures. As an extension to existing methods [6, 17] this method incorporates a regression component of additional correlated data streams. We appraise the following two additional data streams; the time-series of the number of Intensive Care Unit (ICU) admissions and reported cases. The disease stages (infected, hospital, ICU, death) have a time order and the number of new entries in one of the earlier compartments can help estimate what will happen for the later stages. As the additional data streams are assumed to be ahead in time of the fatalities, we use them as leading indicators for the event of interest.

In this paper, we present methodological details of our approach and compare the results to existing nowcasting methods to illustrate the implication of incorporating additional data streams associated with the number of fatalities. We show with a retrospective evaluation of our method that nowcasting with leading indicators can improve performance compared to existing methods.

Materials and methods

Data

The surveillance data used for the analysis in this paper are daily counts of fatalities and ICU admissions and reported cases of people with a laboratory-confirmed SARS-CoV-2 infection in Sweden. The chosen period ranges from 2020-10-20 to 2021-05-21 and contains 117 reporting days (Tuesday to Friday excluding public holidays). During this period, there were 951 646 reported cases, 4 734 ICU admissions and 8 656 fatalities. The evaluation period covers Sweden's second (alpha) and third wave (delta) of COVID-19-related fatalities. In addition, this period also covers the introduction of vaccination which meant a change in the association between reported cases or ICU admissions and the fatalities. The times series of the number of reported cases, ICU admissions, and deaths can be seen in Fig 2. The figure shows that the rise and fall of the three time series follow a similar time trend, with some time delay, during the first wave. However, in the second wave, the relative association between the fatalities and the other disease stages becomes less substantial, the main reason being the introduction of vaccination starting 2020-12-27 in Sweden.

The data used in our analysis is publicly available from the website of the Public Health Agency of Sweden [4], where new reports have been published daily from Tuesday to Friday (excluding public holidays). The aggregated daily counts are updated retrospectively at each reporting date. As the case fatalities are associated with a reporting delay, this implies that the published time series of reported COVID-19 fatalities will always show a declining trend (see Fig 1 for an illustrative example). The reporting delay can not be observed in a single published report but can be obtained by comparing the aggregated numbers of fatalities of each date from previously published reports.

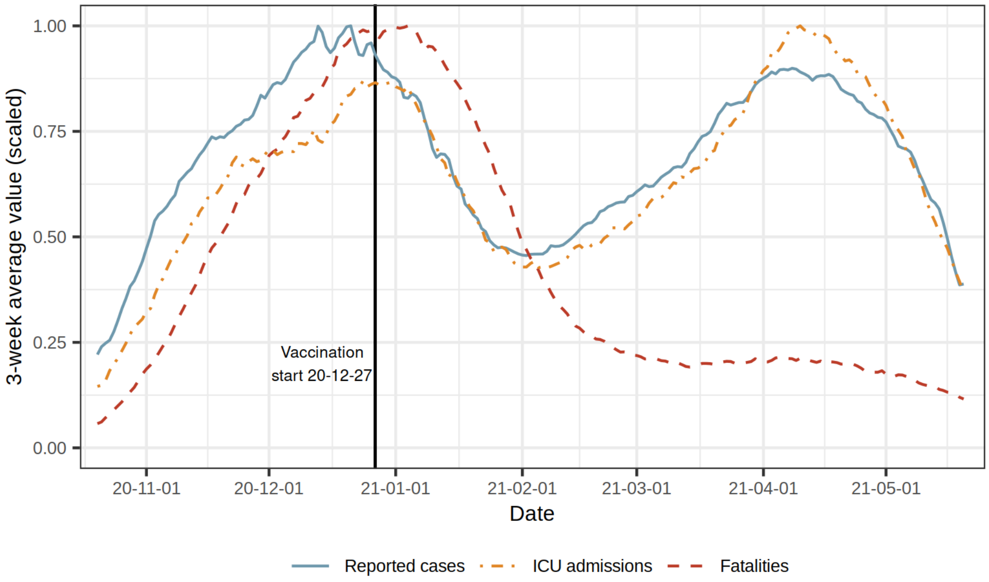

Fig 2. Reported cases, ICU admissions and fatalities with COVID-19 in Sweden. The period covers the second (alpha) and third (delta) wave and the start of vaccination in Dec 2020. Each time series is shown with a 3-week centered rolling average and scaled by its maximum value.

Nowcasting

The notation and methodological details of our approach follows closely the notation introduced in Günther et al. [17]. Let $n_{t,d}$, be the number of fatalities occurring on day $t = 0, ..., T$ and reported with a delay of $d = 0, 1, 2, ...$ days, such that the reporting occurs on day $t + d$. The goal of Nowcasting is to infer the total number of fatalities N_t of day t based on the information available on the current day $T \geq t$. The sum N_t can be written as

$$N_t = \sum_{d=0}^{\infty} n_{t,d} = \sum_{d=0}^{T-t} n_{t,d} + \sum_{d=T-t+1}^{\infty} n_{t,d},$$

where the first sum is observed and the second sum is yet unknown. This can be illustrated by the so called reporting triangle (Fig 3). Where the upper left triangle are the number of reported fatalities and the lower right triangle is the number of occurred-but-not-yet-reported events with a maximum delay of D days. The upper triangle carries the information about the reporting delay from the past and the lower triangle is what is estimated with the Nowcasting model.

We let λ_t denote the expected value of N_t, and $p_{t,d}$ denote the conditional probability of a fatality occurring on day t being reported with a delay of d days. Then, the number of events occurring on day t with a delay of d days is assumed to be negative binomial distributed

$$n_{t,d}|\lambda_t, p_{t,d} \sim \text{NB}(\lambda_t \cdot p_{t,d}, \phi),$$

with mean $\lambda_t \cdot p_{t,d}$ and overdispersion parameter ϕ. Hence, the Nowcasting task can be seen as having two parts; (1) determine the expected value of the total number of fatalities and (2) determine the reporting delay distribution to subsequently predict the $n_{t,d}$'s and finally compute the N_t's.

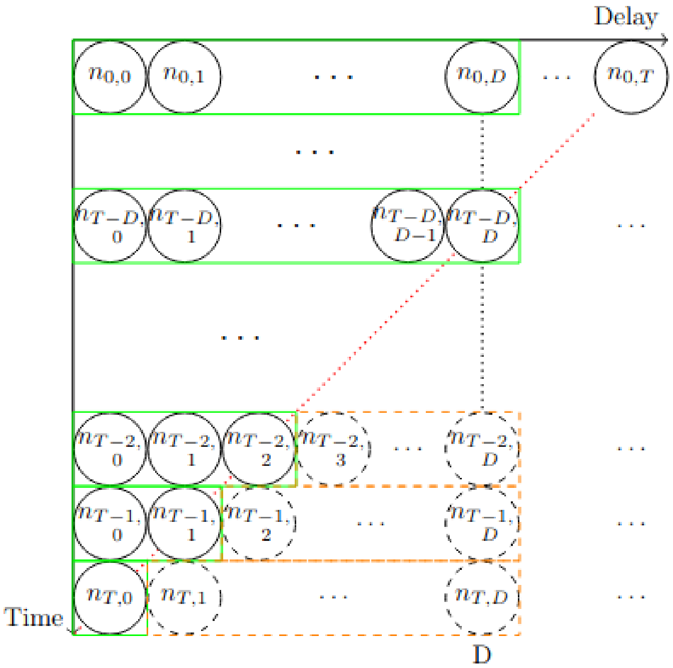

Fig 3. Reporting triangle for day *T*. Green boxes (solid line) where $t \leq T - D$ are the reported number of fatalities on day T (today), considering a maximum delay of D days. The red boxes (dashed line), corresponding to $t > T - D$, are the occurred-but-not-yet-reported number of events of day $t + D$.

Flexible Bayesian Nowcasting

As described in the previous section, the nowcasting problem can be seen as a problem of the joint estimation of two models: (1) a model for the expected number of deaths over time, and (2) a model for the reporting delay distribution, which can also vary over time. Therefore, we let our model constitute of two distinct elements; (1) the underlying epidemic curve determining the expected number of fatalities λ_t and (2) the reporting delay distribution determining $p_{t,d}$. We will in the following describe the structure of each.

Component 1: The expected number of fatalities

Let $\lambda_t = \mathbb{E}[N_t]$ denote the expected total number of fatalities occurring on day t. We specify a baseline model for λ_t as

$$\log(\lambda_t)|\lambda_{t-1} \sim N(\log(\lambda_{t-1}), \sigma^2), \tag{1}$$

where $t = 0, ..., T$ and $d = 0, ..., D$. Time $t = 0$ is assumed to be the start of the chosen observation period, e.g. the start of the pandemic. This approach to model λ_t as a Random Walk on the log scale is proposed by McGough et al. [7] and Günther et al. [17]. Here, we will refer to it as model R.

An alternative to model R in Eq (1) is to assume that we can predict the total number of fatalities with additional data streams associated with the event of interest. The additional data streams are assumed to be ahead in time compared to the time series of interest, e.g. due to the tracked event of the stream being at an earlier stage in a typical COVID-19 disease progression or because of a smaller reporting delay; e.g. the number of reported cases and hospitalizations, etc. Hence, we can use the additional

data stream as a leading indicator in the Nowcasting model. One approach is to consider the number of fatalities as some time-varying fraction of the numbers in those additional data streams. We denote the i'th of the k leading indicators at time t as $m_{i,t}$ and specify an regression type model for λ_t as follows:

$$\log(\lambda_t)|m_{1,t}, ...m_{n,t} \sim N\left(\beta_0 + \sum_{i=1}^{k} \beta_i m_{i,t}, \sigma^2\right), \quad (2)$$

where the β_0 is an intercept and β_i denotes the additive effect of the i'th stream on the log of the mean of λ. With this model specification, we assume a strong association between the case fatalities and the k data streams suitably measured some days earlier. We will refer to this model as L(m_i).

Furthermore, we propose another approach combining the random walk component of the model in Eq (1) and the additional data streams of Eq (2). Here, we let the leading indicators be the relative change in e.g. case reports or hospitalizations. In other words, we assume that if there is an increase in the leading indicator, we also expect an increase in the number of fatalities. An increase in case reports is not expected to give an instant increase in the number of deaths but rather with some time delay, so as for the model in Eq (2), the leading indicators need to be specified with a suitable time delay. We specify this alternative model for λ_t as

$$\log(\lambda_t)|\lambda_{t-1}, m_{1,t}, ..., m_{n,t} \sim N\left(\log(\lambda_{t-1}) + \sum_{i=1}^{k} \beta_i m_{i,t}, \sigma^2\right), \quad (3)$$

where the β_i's are again considered as regression coefficients for the leading indicator m_i. This approach combines an established method [17] with additional information that is informative of the events of interest. We note that when the β-coefficients of this model are zero, this model becomes identical to the model specified in Eq (1). This model will be referred to as RL(m_i).

Component 2: The reporting delay distribution

The model for the reporting delay distribution at day t is specifying the probability of a reporting delay of d days for a fatality occurring on day t. We denote this conditional probability

$$p_{t,d} = P(\text{delay} = d|\text{fatality day} = t).$$

Similarly to Günther et al. [17], we model the delay distribution as a discrete time hazard model $h_{t,d} = P(\text{delay} = d|\text{delay} \geq d, W_{t,d})$ as

$$\text{logit}(h_{t,d}) = \gamma_d + W'_{t,d}\eta, \quad (4)$$

where $d = 0, ..., D-1, h_{t,D} = 1$, γ_d is a constant, $W_{t,d}$ being a vector of time- and delay-specific covariates and η the covariate effects. It can be shown how the reporting probabilities are derived from Eq (4) [17]. We are using linear effects of the time on the logit-scale with break-points every two weeks before the current day to allow for changing dynamics in the reporting delay distribution over time. We also use a categorical weekday effect to account for the weekly structure of the reporting.

Inference and implementation

Inference for the hierarchical Bayesian nowcasting model is done by Markov Chain Monte Carlo using R-Stan [19] extending the work of Günther et al. [17]. In order to ensure reproducibility and transparency, the R-Code [20] and data used for the analysis is available from `https://github.com/fannybergstrom/nowcasting_covid19`.

Results

Application to fatalities

We apply the nowcasting methods to reported COVID-19 fatalities in Sweden and let the number of reported cases and COVID-19 associated ICU admissions act as two leading indicators. The reporting of ICU admissions is also associated with a reporting delay but considerably shorter than the fatalities. We use model R as a benchmark model and compare it the two alternative models using leading indicators; model L where we let the leading indicator be the number of COVID-19-related ICU admissions, and model RL including both the random walk component and leading indicator here being the relative weekly change in ICU admissions. We denote the leading indicator models as L(ICU) and RL(ICU). For the leading indicator time series, we use a seven day centered rolling average to avoid the weekday effect of the reporting. The pre-specified lag between the fatalities and leading indicators is determined by fitting a linear time series model given the two model specifications of models L and RL and choosing the lag providing the best fit. The period chosen for the time series model is 2020-04-01–2020-10-19 to use the information available only prior to the evaluation period. We use 18 days lag for the reported cases and 14 days for the ICU admissions. The reporting probability is set to be zero on non-reporting days (Saturday–Monday and public holidays). For practical and robustness reasons, we use a maximum reporting delay of $D = 35$ days. For the fatalities reported with a longer than the maximum, we set their delay to the upper limit of 35 days. There were 116 case fatalities reported with a delay longer than 35 days during the evaluation period.

Retrospective nowcasting evaluation

We use a retrospective evaluation in order to assess the performance of the Nowcasting models. The model-based predictions are compared to the (now assumed to be known) final number of COVID-19-related reported fatalities in Sweden. The samples from the posterior predictive distribution for the total number of reported COVID-19 fatalities $\hat{N}_t$ are extracted for each reporting date of the evaluation period. As in Günther [17], we use the following four metrics to quantify the model performance; 1) continuous rank probability score (CRPS), 2) log scoring rule (logS), 3) root mean squared error (RMSE), and 4) the prediction interval (PI) coverage being the proportion of times the true number of fatalities is contained within the equitailed PI. The RMSE is calculated with a point estimate being the median of the posterior predictive samples of $\hat{N}_t$, while the scoring rules CRPS and logS assess the quality of the probabilistic forecast by considering the full posterior distribution of $\hat{N}_t$ [21]. For the scoring rules, a low score indicates a better performance.

Nowcasts and the estimated reporting delay for a specific reporting date T=2020-12-31, is shown in Fig 4. In the left column, the black bars are the number of fatalities reported until day T and the red dashed line is the true number, only known in retrospect. The solid lines are the median of the posterior predictive distribution of $\hat{N}_t$ and the shaded areas indicate the equitailed point-wise 95% Bayesian prediction interval, estimated with information available at the reporting date. The right column shows the daily empirical and estimated number of days of reporting. The solid lines are the estimated and empirical median days of reporting delay and the shaded area is between the 5% and 95% quantile of the reporting delay. The lower bound indicate the number of days until 5% of the total number of fatalities will be reported and the upper bound is within how many days 95% will be reported. The empirical median and the respective quantiles are calculated with data available in hindsight and the estimated quantities are obtained with the information available at the reporting date.

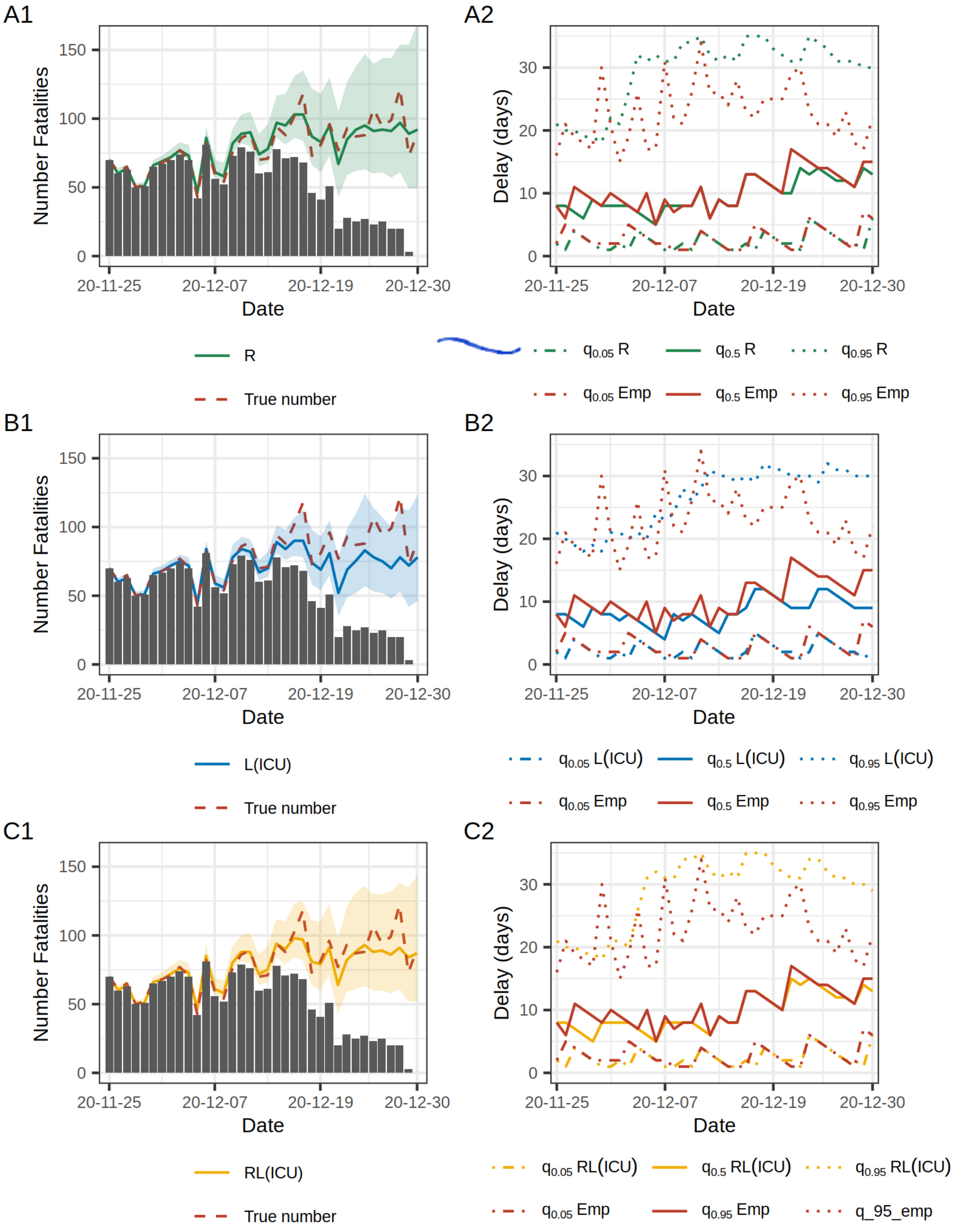

Fig 4. Nowcasts for a specific reporting date. Left column shows the nowcasts of 2020-12-30, where the solid lines are the median of the posterior predictive distribution of $\hat{N}$ and the shaded area depict the 95% PI. The black bars are what is yet reported and the red line is the true number, only known retrospectively. Right column shows quantiles of the estimated and empirical reporting delay distribution. The solid lines the median reporting the delay in days (for each date) and the lower and upper bounds are the 5% and 95% quantiles. At the 5% quantile, 5% of the total number of fatalities occuring on that date are estimated to be reported within the number days delay, ect. The empirical quantiles are obtained with data available in hindsight.

We observe an underestimation of the reporting delay for the L(ICU) model for the last days in the observation window (2020-12-25–2020-12-30) resulting in an underestimation of the daily number of fatalities (Fig 4B). We can also note that the PI is more narrow for L(ICU) than for the other two models and that the true number is not always contained in the PI. Model R and RL(ICU) (Fig 4A & C) provide similar results with less underestimation of the reporting delay resulting in a point estimate of the median of the predictive distribution lying closer to the true number compared to model L(ICU). A difference between the performance between R and RL(ICU) is that RL(ICU) provides less wide PI than R. For R and RL(ICU), the true number of daily fatalities is contained in the PI for all days T-t, $t = 0, \dots, 35$. From the right column of the figure, it can be observed that the 5% quantile of the estimated number of days of reporting delay for all three models are similar to the empirical 5% quantile. Also, the median of the estimated number of days delay follows the corresponding empirical quantity reasonably well. Contrary, the 95% estimated quantiles are farther from the empirical. This indicate that all three models capture the short-term trends such as the weekly reporting patterns well but do not fully capture the changing dynamics of the long reporting delays, i.e. the high spikes in the early observation window and the rapid decrease in the final week. An alternative visualization of the empirical and estimated reporting delay distribution for the three models provided by the cumulative reporting probability is found in S1 Appendix Sec 1.

Seen in Fig 4, the PI is increasing as the final date T of the observation window is approaching. As the number of days t since day T decrease, the uncertainty for the nowcast of day T-t increase as the fraction of the reported fatalities will be decreasing. The average score as a function of T-t is shown in Fig 5. For all models and scores, the score is generally a decreasing function of the number of days since day T. Hence, the farther from "now", the closer are the nowcast estimates of the daily number of fatalities to the true number. The difference in performance for the three models is observable for the two weeks prior to day T. Here, we see that model RL(ICU) has a lower CRPS and RMSE score (Fig 5A & C) and that model R has the lowest logS (Fig 5B). Model L(ICU) has the overall highest values of the scores, hence it has the worst performance of the three models.

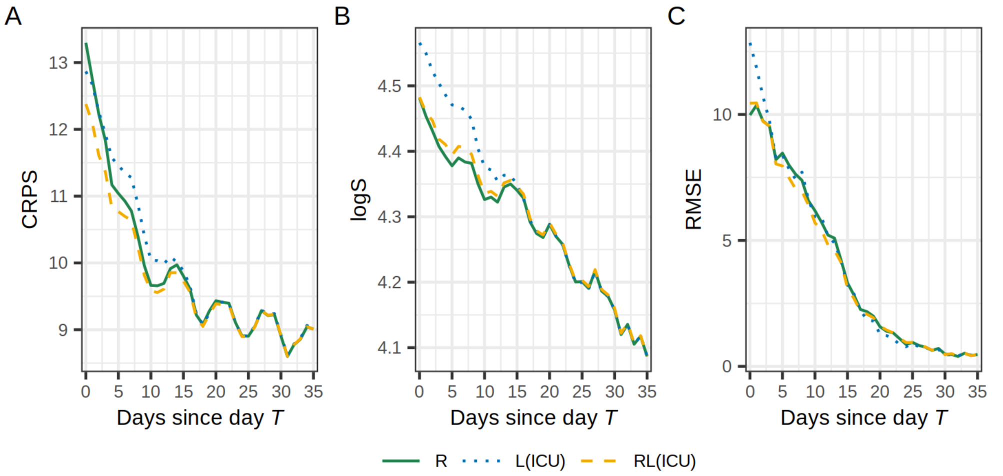

Fig 5. Mean scores by the number of days since the day of reporting *T*. The results are averaged over all reporting dates T in the evaluation period from 2020-10-20 to 2021-05-21.

The mean overall score and the coverage frequency of the 75%, 90%, and 95% prediction interval of the three models for the nowcasts performed in the evaluation period is found in Table 1. For each reporting day T, we consider the average score of the last seven days; $T-6,\ldots,T-0$. Based on the CRPS and RMSE, model RL(ICU) has the best performance, with a decrease of 4.2% and 1.0% respectively compared to model R. Model R has the lowest logS score but only with a slight advantage compared to RL(ICU) (0.02% improvement). Model L(ICU) has the worst performance for all three scores. The coverage of the prediction intervals for models R and RL(ICU) is of satisfactory levels. In contrast, the L(ICU) model has low coverage, indicating that model L(ICU) is less trustworthy.

Table 1. Results of the retrospective evaluation of different nowcasting models on COVID-19 related fatalities in Sweden.

Score	R	L(ICU)	RL(ICU)
CRPS	11.89	12.01	**11.39**
logS	**4.42**	4.51	4.43
RMSE	9.18	9.95	**9.09**
Cov. 75% PI	76.07%	58.97%	76.07%
Cov. 90% PI	95.72%	76.07%	94.87%
Cov. 95% PI	98.29%	84.61%	99.14%

CRPS is the continuous ranked probability score, logS is the log score, and RMSE denotes the root mean squared error of the posterior median. Additionally, we provide coverage frequencies of 75%, 90% and 95% credibility intervals in the estimation of the daily number of case fatalities. The scores are averaged over nowcasts for day $T-6,...,T-0$, with T being all reporting dates in the evaluation period.

Fig 6 shows the retrospective true number of daily fatalities and the median of the predictive distribution of $\hat{N}$ and a 95% PI of day T for the three models evaluated on each reporting day in the evaluation period. In Fig 4, this corresponds to the nowcast estimates of the final date T=2020-12-30. We observe a similar performance over time for models R and RL(ICU) (Fig 6A & C) and the more significant deviations from the true number appear mainly on the same reporting dates for the two models. In early Jan 2021, RL(ICU) underestimates the number of daily fatalities, likely due to the rapid decrease in ICU admissions due to the introduction of vaccines at the end of Dec 2020, while the case fatalities were also on a downwards trend but not as steep. Model RL(ICU) stabilizes after approximately two weeks (same as the length of the linear change points) in mid Jan 2021 as the model adapts to the new association between ICU admissions and case fatalities. Model L(ICU) (Fig 6B) does not have the high peaks in the posterior predictive distribution of $\hat{N}$ as the other two models. However, the deviation of the posterior median compared to the true number is visibly larger. Starting from Dec 2020, we observe an underestimation of the number of fatalities, and from Feb 2021, an overestimation for the following two months. From Apr 2021 until the end of the evaluation period, the three models have a visibly similar performance with a posterior mean close to the true number of daily fatalities and a narrow PI containing the true number.

The performance of the alternative models with leading indicators compared to model R can be explained by the estimated association between the fatalities and the leading indicators. The changing dynamics of the association over time are captured by the estimated time-varying β-coefficients of the respective models. Details of the estimated β-coefficients for models R(ICU) and RL(ICU) over the evaluation period are reported in S1 Appendix Sec 2.

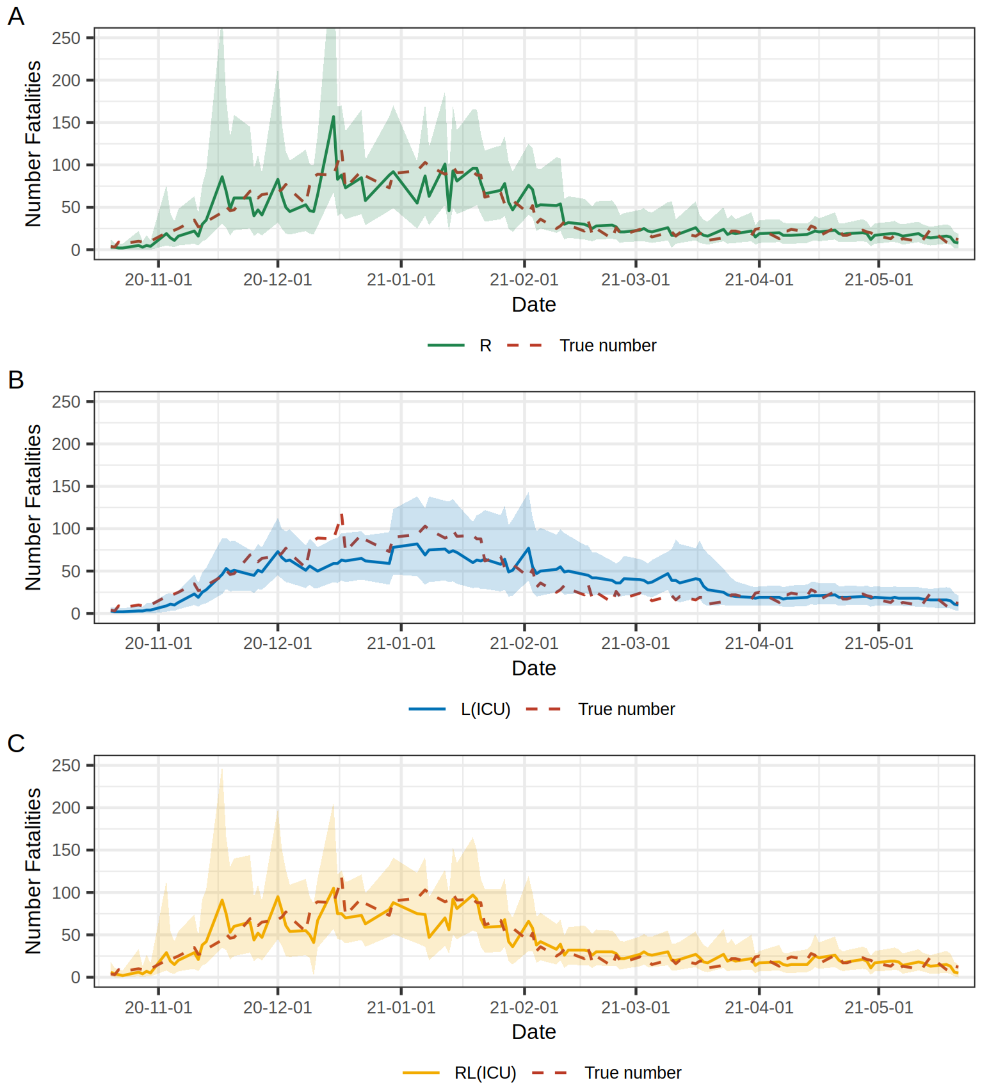

Fig 6. Estimated and true number of fatalities with COVID-19 in Sweden. The estimated number of fatalities are the nowcasts of day T being each reporting date in the evaluation period from 2020-10-20 to 2021-05-21. The solid lines are the median of the posterior predictive distribution of the number of daily fatalities $\hat{N}_T$ and the shaded area depict the point-wise 95% PI. The red line is the retrospective true number.

The scores of the three models evaluated at the 117 reporting dates in the evaluation period by the CRPS and LogS is shown in Fig 7. For each reporting day T, we consider the average score of the last seven days; $T-6, \ldots, T-0$. For the three models, the scores are generally higher when the number of case fatalities is high. Overall, the performance of model R and RL(ICU) is similar, as could also be observed in Fig 6. From the beginning of the evaluation period until the end of 2020, model L(ICU) has an overall lower score and a more stable performance with less high spikes in the score compared to model R and RL(ICU). During Jan 2021, the performance is similar for the three models, but from Feb to Apr 2021 model L(ICU) performs significantly worse than the other models. The remaining scoring rule, the RMSE, entail similar results (S1 Fig). After Apr 2021, the number of daily fatalities has stabilized to a low number and

the score for three models becomes similar until the end of the evaluation period.

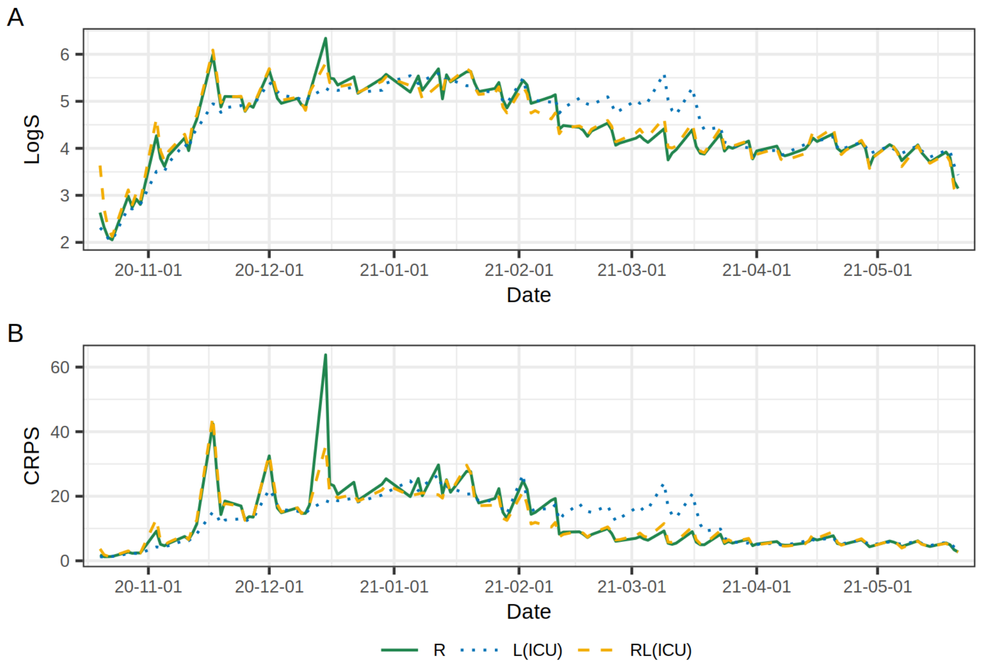

Fig 7. Scoring rules. Average CRPS and logS of the last 7 days; $T-6,\ldots,T-0$ for each reporting day T, in the evaluation period.

In conclusion, we find that model R and model RL(ICU) performs well over the evaluation period and has a satisfactory level of PI coverage. Furthermore, model RL(ICU) provided the best performance of the three models, indicating that there is a gain (4.2% decrease in CRPS compared to model R) of including leading indicators. Using reported cases or the combination of reported cases and ICU admissions as leading indicators does not improve performance. The results of using these leading indicators are found in S1 Appendix Sec 3.

Discussion

In the presented work, we provide an improved method for real-time estimates of infectious disease surveillance data suffering a reporting delay. The proposed method can be applied to any disease for which the data can be put the form of the reporting triangle given in Fig 3. We apply the method to COVID-19-related fatalities in Sweden. Even though fatalities are a lagging indicator to obtain situational awareness about the pandemic and is not without difficulties itself, it is often used as a more robust indicator to assess the burden of disease because it might be less influenced by the current testing strategy. Hence, monitoring the time series of reported deaths has been of importance in the still on-going COVID-19 pandemic.

We show that using leading indicators, such as the COVID-19-associated ICU admissions, can help improve the nowcasting performance of case fatalities compared to existing methods. Beyond using reported cases and ICU admissions as leading indicators for the case fatalities, other possible leading indicators are e.g. vaccination, hospitalizations, and virus particles in wastewater [22], or using age-stratified reported cases. However, nowcasting with leading indicators should be made with caution and be

reevaluated as the dynamics between the leading indicator and the event of interest change, which may not be a trivial task during an ongoing pandemic. Furthermore, by re-estimating the association coefficients of the leading indicator at each reporting date, our method captures the changing association between ICU admissions and case fatalities over time. However, we use a pre-specified time lag unknown at the start of the pandemic and might also change throughout the pandemic. A possible extension of our work would thus be to estimate this time lag as a part of the model fitting.

The proposed method is flexible in terms of its application and thus can be a helpful tool for future pandemic stress situations. We support this by providing open-source software for the real-time analysis of surveillance data. Weekly updated nowcast estimates of COVID-19 fatalities and ICU admissions in Sweden using our proposed method, model RL, are found at

https://staff.math.su.se/fanny.bergstrom/covid19-nowcasting

These graphs help provide the desired situational awareness and are to be interpreted as new variants emerge.

Supporting information

S1 Fig. RMSE. Average RMSE of the last 7 days; $T-6,\ldots,T-0$ for each reporting day T, in the evaluation period.

S1 Appendix. Complimentary material and results. Sec 1 contains information about the cumulative reporting probability, providing a complimentary picture of the estimated reporting delay. Sec 2 presents detailed results of the estimated regression coefficients of model L(ICU) and RL(ICU) over the evaluation period. Finally, Sec 3 covers results of including reported cases and the combination of reported cases and ICU admissions as leading indicators.

Acknowledgments

We thank Markus Lindroos for discussions and his contribution in coding of the reporting delay distribution.

References

1. Metcalf C, Morri D, Park S. Mathematical models to guide pandemic response: Models can be used to learn from the past and prepare for the future. Science. 2020;369(6502).

2. Wu JT, Leung K, Lam T, Ni MY, Wong C, Peiris J, et al. Nowcasting epidemics of novel pathogens: lessons from COVID-19. Nat Med. 2021;27:388–395.

3. Lawless JF. Adjustments for reporting delays and the prediction of occurred but not reported events. Can J Stat. 1994;22(1):15–31.

4. Folkhälsomyndigheten. The Public Health Agency of Sweden's COVID-19 data portal; Accessed 2022-03-07. https://www.folkhalsomyndigheten.se/smittskydd-beredskap/utbrott/aktuella-utbrott/covid-19/statistik-och-analyser/.

5. Donker T, van Boven M, van Ballegooijen WM, Van't Klooster TM, Wielders CC, Wallinga J. Nowcasting pandemic influenza A/H1N1 2009 hospitalizations in the Netherlands. Eur J Epidemiol. 2011;26(3):195–201.

6. Höhle M, an der Heiden M. Bayesian nowcasting during the STEC 0104:H4 outbreak in Germany, 2011. Biometrics. 2014;70:993–1002.

7. McGough SF, Johansson MA, Lipsitch M, Menzies NA. Nowcasting by Bayesian Smoothing: A flexible, generalizable model for real-time epidemic tracking. PLOS Comp Bio. 2020;16(4):e1007735.

8. Kaminsky KS. Prediction of IBNR claim counts by modelling the distribution of report lags. Insurance: Mathematics and Economics. 1987;6:151–159.

9. Kalbfleisch J, Lawless JF. Inference based on retrospective ascertainment: an analysis of the data on transfusion-related AIDS. JASA. 1989;84(406):360–372.

10. Zeger SL, See LC, Diggle PJ. Statistical methods for monitoring the AIDS epidemic. Stat Med. 1989;8(1):3–21.

11. Greene S, McGough S, Culp G, Graf L, Lipsitch M, Menzies N, et al. Nowcasting for real-time COVID-19 tracking in New York City: Evaluation study using reportable disease data from the early stages of the pandemic. JMIR Public Health and Surveillance. 2021;7.

12. Li T, White LF. Bayesian back-calculation and nowcasting for line list data during the COVID-19 pandemic. PLOS Comp Bio. 2021;17(7):1–22.

13. Shaun R, Pantelis S, Meaghan K, De Angelis D. Nowcasting COVID-19 deaths in England by age and region. J R Stat Soc Series C. 2022;1–16.

14. Schneble M, De Nicola G, Kauermann G, Berger U. Nowcasting fatal COVID-19 infections on a regional level in Germany. Biom J. 2020;63(3):471–489.

15. Altmejd A, Rocklöv J, Wallin J. Nowcasting COVID-19 statistics reported withdelay: a case-study of Sweden; 2020. Available from: https://arxiv.org/abs/2006.06840.

16. Bird S, Nielsen B. Now-casting of COVID-19 deaths in English hospitals. University of Oxford.; 2020. http://users.ox.ac.uk/~nuff0078/Covid/.

17. Günther F, Bender A, Katz K, Küchenhoff H, Höhle M. Nowcasting the COVID-19 pandemic in Bavaria. Biom J. 2020;63(3).

18. Peng Y, Chen X, Rong Y, Pang C, Chen X, Chen H. Real-time Prediction of the Daily Incidence of COVID-19 in 215 countries and territories Using Machine Learning: Model Development and Validation. JMIR. 2021;23.

19. Stan Development Team. RStan: the R interface to Stan; 2020. Available from: http://mc-stan.org/.

20. R Core Team. R: A Language and Environment for Statistical Computing; 2021. Available from: https://www.R-project.org/.

21. Gneiting T, Raftery A. Strictly Proper Scoring Rules, Prediction, and Estimation. JASA. 2007;102:359 – 378.

22. Kreier F. The myriad ways sewage surveillance is helping fight COVID around the world [published online ahead of print, 2021 May 10]. Nature. 2021;10.1038/d41586-021-01234-1.

Click here to access/download;Figure;S1_fig.png

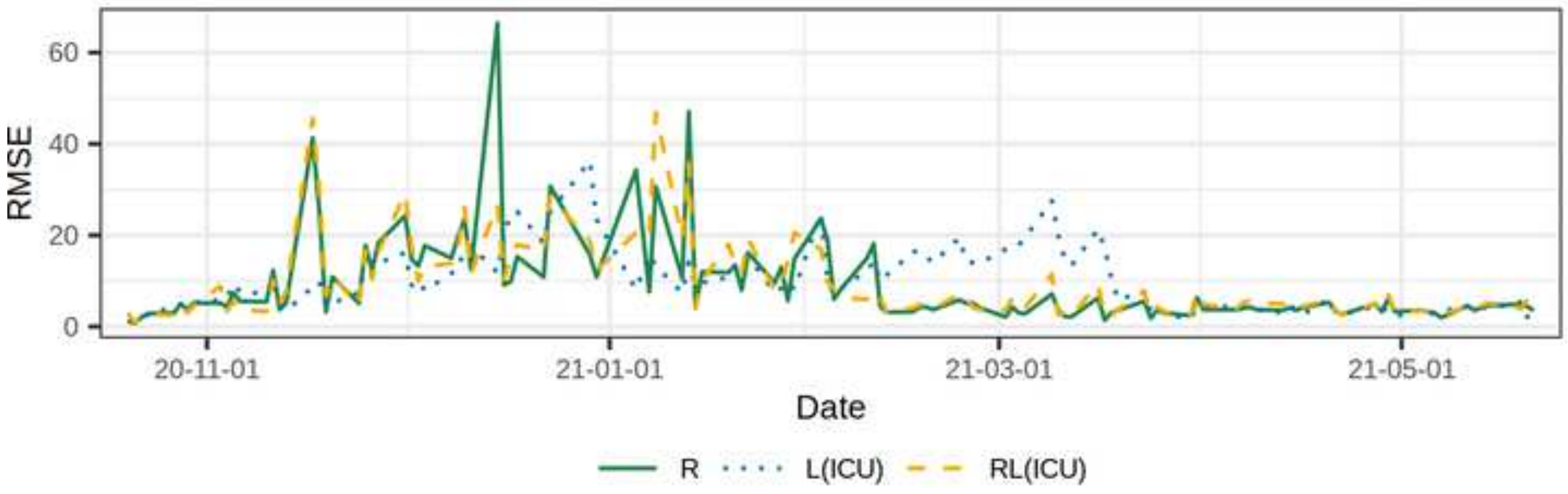

Click here to access/download
Supporting Information
SI_Appendix_Nowcasting_COVID19.pdf
