# Supplementary material for: Bayesian nowcasting with leading indicators applied to COVID-19 fatalities in Sweden
Source: PLoS Comput Biol. 2022 Dec 7;18(12):e1010767. doi: 10.1371/journal.pcbi.1010767 (PMC9762573; doi:10.1371/journal.pcbi.1010767)
Supplement: Attachment — Submitted filename: Response_Letter.pdf [file pcbi.1010767.s004.pdf]

Fanny Bergström
Department of Mathematics, Stockholm University
+46 709694640
fanny.bergstrom@math.su.se

To
Claudio José Struchiner, M.D., Sc.D.
Academic Editor
PLOS Computational Biology

November 11, 2022

Rob De Boer
Senior Editor
PLOS Computational Biology

Resubmission: Bayesian Nowcasting with leading indicators applied to COVID-19 fatalities in Sweden.

Dear Claudio José Struchiner and Rob De Boer,

Hereby we resubmit our research article entitled *Bayesian Nowcasting with leading indicators applied to COVID-19 fatalities in Sweden* for publication in PLOS Computational Biology. We are grateful for the review comments and have addressed these in the revised version. Furthermore, you find our point-to-point response to the reviewer's comments below, starting with general comments and then addressing all points given by the reviewers. Italic text are the reviewers comments and the blue text is our response.

Please also note that, while working on the revision we found a minor inaccuracy in the calculation of the evaluation metrics. The calculations of the CRPS and logS are now optimized and some inadvertently left-out observations in the calculations of the PI coverage are now included. This means that the absolute numbers in the result tables and figures of the scores have changed slightly, but the relative performance between the three models remains similar and the overall conclusions of the results are thus unchanged.

We thank the three reviewers for their thoughtful comments and suggestions which has improved our manuscript. We look forward to your reply.

Best regards,

Fanny Bergström

(on behalf of all authors; Felix Günther, Michael Höhle, and Tom Britton)

Point-to-point Reply

Both reviewer 1 and 2 pointed us to a reference of a similar nowcasting model by Bastos et al. (2019). We thank you for this and agree that this citation is of importance for our work. It is now included in the revised manuscript and we relate it to our work by a theoretical comparison between our model and the model by Bastos et al. (SI Appendix Sec 7). The Github repository is now also accessible at `https://github.com/fannybergstrom/nowcasting_covid19`.

Reviewer 1

I read with great interest the manuscript PCOMPBIOL-D-22-01107, "Nowcasting with leading indicators applied to COVID-19 fatalities in Sweden". The authors extended the method described in Günther et al. (2020) adding the possibility to include covariates in to the model. The inference procedure is done via MCMC and the authors have implemented their model in R-Stan. Their motivation was to provide delay corrected estimates for the daily number of deaths due to COVID-19 in Sweden. I think it is an important topic and I would like to comment some points that I think should be considered in their manuscript. The timing of the manuscript is interesting as well, since the number of cases and deaths due to COVID is increasing now in Sweden.

There is a very similar nowcast model based on the chain-ladder model that already takes into account covariates and also spatial random effects in the mean component. However, the aforementioned paper is pre COVID where the authors apply their method on dengue fever and on severe acute respiratory illness (SARI), Bastos et al. (2019). Miller et al. (2022) use that method to correct delays of Chikungunya fever notification in Brazil by using Google searchers and Tweets to improve the nowcasting estimates. The point here is that incorporating regression components in this class of nowcasting models is not the main novelty here, but having said that the use of such methods to improve estimates of COVID-19 fatalities is very important and worth exploring.

Thanks for pointing us to this reference, which we should have known. The two papers are now included in our introduction. We now also refer to the model by Bastos et al. (2019) in the Sec Materials and Methods (on line 135) and provide a theoretical comparison between this and our model in the Appendix Sec 7.

The authors should explain more how the delay was calculated. Since there are some days as I understood which new datasets are not provided (weekends and bank holidays) So there may be some "holes" in the matrix described in Fig. 3. For some lines there would not have values for certain columns. For example, if day t

We have added further explanation of how the reporting delay is calculated in the subsection Component 2: The reporting delay distribution (on line 146). It is correct that the reporting triangle has diagonal lines of no reporting. We have added a figure of the reporting triangle of the Swedish reporting to illustrate this in the Appendix Sec 2 which we refer to on line 240 in the manuscript. The comment of the referee was not finalized but we hope that we interpreted the referee's comment correctly.

The author present three models, model R where log(λ_t) follows a first order random walk, model L(m_i) where log(λ_t) doesn't depend directly on the past but there are k-leading covariates and model RL(m_i) combining both. Is the computation time similar among them? Of course that would depend on the dimension of (m_i).

We have added running times for the models in the Appendix Table S2 when performing nowcasts for day T=2020-12-30. Running times for this reporting date are 107 s for model R, 62 s for L(ICU) and 288 s for RL(ICU) and as the reviewer also suspects: the running time depends on the number of

leading indicators.

Is this model fast? In Bastos et al. (2019) R-INLA was used because an MCMC approach would be too timely consuming and that wouldn't be efficient on a large surveillance system (a MCMC approach was implemented on NIMBLE and the computational cost of the two approaches was very clear). In this manuscript the authors have implemented their approach in RStan which is good idea since Stan is faster than other MCMC softwares and require less iterations due to the implemented Hamiltonian Monte Carlo with the No-U-turn sampler (NUTS).

Yes, model inference is reasonably fast for our purpose (see above answer, i.e. running times below 1-5 minutes). Given the current running times, the speed benefits of an INLA implementation vs. the cost of making it seems to speak against such an endeavor. Furthermore, due to our two-component hierarchical construction, implementing this in INLA also does not seem straightforward without deep-diving into the numerics of INLA. Hence, we appreciate the suggestion, but due to the different model approaches we instead provide a theoretical comparison showing that our approach is not directly translatable to a GLM, which also hints at an INLA implementation being slightly complicated.

I though that providing an website with the most up-to-date results was quite clever, specially now with an increase of cases and deaths in Sweden. However I couldn't access the code on github page indicated in the manuscript (https://github.com/fannybergstrom/nowcasting_covid19), I believe the repository is still private.

The repository is now public, we apologize for this glitch.

An overall comparison between Bastos et al. approach and the proposed approach would be interesting, but in my humble opinion not really required for this paper. Comparing all different available nowcasting methods for deaths due to COVID-19 would be a very interesting paper, but I believe it is beyond the scopus of this manuscript that focus on COVID fatalities in Sweden.

We agree that this overall empirical comparison would be interesting, but beyond the scope of the manuscript, as our focus was on introducing a new method. However, we did add this to the Discussion (line 407). We have also added a comparison with the Bastos et al. (2019) work in the Appendix, which shows the theoretical differences of the two approaches.

A description of the scoring rules used for retrospective evaluation of the nowcasts should be presented in the Materials and Methods section. Quoting Bracher et al. (2021) "Both the logS and the CRPS cannot be evaluated directly if forecasts are provided in an interval format." perhaps the authors should consider scoring rules that take into account intervals. Comparing the interval coverage may be not enough to represent the uncertainty, since my guess is that by adding a covariate uncertainty of the nowcast estimates would be reduced, i.e. the size of the intervals would be smaller, and that would make a difference since according to the criteria presented in the models seem to behave quite similar, Fig 7 suggests that models L(ICU) and RL(ICU) in general perform better than R model, but it is difficult to decide either RL or L model is better, a measure that quantifies the uncertainty could point out which one stands out.

Thank you for the suggestion. We have added a section describing the scoring rules in the Materials and Methods section. We evaluate the full posterior distribution of $\hat{N}$ which accounts for the uncertainty in the prediction. As the reviewer points out, the performance between model R and RL(ICU) is similar but given the performance measures in Table 1 we find that model RL(ICU) improves the nowcasting performance.

As the authors mentioned the ICU admissions also suffer from delay. The proposal approach seems good since if we take the natural history of the disease there a time between ICU admission until the

death due to COVID, so the ICU delay may be ignored. However, a two-step process could be consider where the R model would be run to ICU data, and then the corrected estimates (ICU) would be used in models L(ICU*) and RL(ICU*). I believe this joint approach could easily be coded in RStan.*

Yes, we agree that this could be implemented RStan. The value of this would, however, be less convincing for our application, because we use the ICU value from two weeks ago, which is pretty complete (96.6% of ICU admissions are available) and does not require a nowcast (this information is now added the section Application to Fatalities on line 230). We do like the idea and can think of situations where this double-nowcasting makes sense, hence, we have also added this to the Discussion (line 389).

In equation (1) and (3) a first order random walk is assumed for log(lambda$_t$), I wonder if a second order random walk would bring smoother estimates and then improve the estimates.

We implemented an AR(2) model for λ_t to investigate if smoother estimates would improve the results. Preliminary results (using 11 reporting dates) showed that there was no improvements over using a simple random walk. We added this to the discussion (on line 391) and the results of the suggested analysis is found in the Appendix Sec 6.

Priors. What prior distributions were used for sigma (random effects variance), beta's (regression coefficients in models L and RL), phi (negative binomial overdispersion parameter) and gamma$_d$ (equation 4). I am assuming the eta parameters (equation 4) were not used in the COVID fatality models right?

Thanks for putting more focus on the choice of priors. The list of prior distributions used in our model is now included in the SI Appendix Table S1. Yes, we do use the eta parameters as given in Eq (4).

Reviewer 2

As usual, since the identity of the authors is known to me, I will be signing this review in the interest of fairness. Best, Luiz Max Carvalho.

Major comments.

In this a well-written paper, Bergstrom and colleagues address the issue of nowcasting COVID-19 in Sweden using a flexible modelling strategy that includes information on ICU admission to produce better nowcasts of case numbers. While I commend the authors for their clear presentation and well-made figures, I would like to point out that the methodology developed on pages 5 and 6 can be considered a special case of the methodology put forth by Bastos et al. (2019, Statistics in Medicine). The omission of this citation is in my opinion a major oversight that needs immediate addressing. Moreover, since methodologically the paper does not add anything new to the state-of-the-art, its merits must lie with its empirical findings. On that front, I am uncertain as to what exactly is the advantage of RL(ICU) compared to R. I suppose it doesn't hurt to include ICU information, as long this is done carefully – look at the performance of L(ICU).

In summary, I regard this as a well-written paper that unfortunately fails to mention a crucial piece of literature and therefore misses the opportunity to improve on the state-of-the-art.

We agree that including the work of Bastos et al. (2019) helps put our work in perspective. Thanks for pointing us to this work. Unfortunately, it appears that due to the sheer volume of COVID-19 related publications, much relevant pre-pandemic work is over-looked. In addition we have included a comparison with that model. See also our answer to Comment 1 by reviewer 1.

Minor comments

These models can be implemented in INLA (https://www.r-inla.org/) which is much faster than

Stan. I appreciate the Stan implementation (i) allows for more complex models to be implemented if desired and (ii) is (probably) plenty fast already. And that is why this is listed as a minor point.

Yes, as we also mention in our answer to reviewer 1, we find the model inference sufficiently fast for our purpose. To address the point of speed we now provide the running times in the Appendix in Table S2.

I really like the use of CRPS for (retrospectively) assessing model predictions. The fact that it is a proper scoring rule should be emphasised more, I think.

We agree and have added a section describing the scoring rules emphasising that these are proper scoring rules (Section Evaluation metrics).

The repository the authors point to for the code does not exist.

The repository is now public, we apologize for this mistake.

I have marked up a few English mistakes/typos/awkward uses. See attached PDF.

Typos/wordings in attached PDF are corrected. Thanks!

Reviewer 3

The authors present a nice expansion of the Nowcasting method introduced by Gunther et al. in the Biometrical Journal in 2021 and apply it to a new data set from Sweden. The introduction effectively motivates the need for generating plausible estimates of the current levels of mortality and ICU admittance given delays in reporting. Figure 1 makes the reporting lag issue very clear. But there are many COVID-19 Nowcasting papers – a quick PubMed search returns 66 results. So what is novel here? Primarily, the authors incorporate leading indicators as covariates and compare performance to the Gunther et al. model and a hybrid of the two. The Gunther et al. model is highly cited, and improvements on this methodology could contribute to better results in the literature moving forward. The statistics used to evaluate model performance are nicely chosen and presented, indicating a slight improvement by using the hybrid approach.

Major comments
My primary challenge in evaluating the updated methodology is understanding Equations 3 and 4 and the number of parameters being estimated in each of the models. Plots in the Supplement indicate time-varying coefficients while the equations do not indicate variation in the coefficient values with a time index. Unfortunately, it appears the repo with the code is currently private so I was unable to evaluate alignment between the described methodology and the actual implementation. I would request that the authors make the repo accessible and allow for reassessment of the new methodology, as the model specification is not entirely clear from the written description. Aside from the need to more closely interrogate the novel model, I only have minor revisions for the authors and believe that with a clearly understanding of the core equations I will enthusiastically recommend acceptance.

We thank the reviewer for the positive comments and apologize about the GitHub repository not being public. This is now fixed. We have added some further explanation in the Supplement (Sec 4) describing the figure of the estimated β-coefficients over time and we hope that this together with the open source code will enable a full understanding of the methodology. We have also updated the formulations of Equations (2)-(3) given your comment below regarding the matrix notation and Equation (4) after reviewer 1's comment about the reporting delay calculations. We believe that these updates of the core equations of our method facilitates the understanding of the models.

Minor revisions
- *Line 17: "Nowcasting methods _have_ been used"*
- *Line 18: No comma*
- *Line 22-23: Revise for clarity*

- *Fig 2: Recommend scaling to max value in first peak to see the relationship between all three before vaccines are introduced*
- *Fig 3: Red box is looking orange*
- *Equations 2 and 3: I would use the matrix notation with a capital M to align with equation 4 and shift the model naming convention to be L(M) and RL(M)*
- *Equation 4: Is W a vector or a matrix? It is a vector and is now clarified on page 6 line 144.*
- *Line 169: Consider using a percent of the total instead of a count*
- *Line 186: "_are_shown in Fig 4"*

Thanks for the suggestions. All minor comments are addressed.
